# The Proteasome and Cul3-Dependent Protein Ubiquitination Is Required for Gli Protein-Mediated Activation of Gene Expression in the Hedgehog Pathway

**DOI:** 10.3390/cells13171496

**Published:** 2024-09-06

**Authors:** Tomasz Uśpieński, Paweł Niewiadomski

**Affiliations:** Centre of New Technologies, University of Warsaw, Banacha 2c, 02-097 Warsaw, Poland

**Keywords:** Hedgehog signaling, Hedgehog signalling, Gli proteins, proteasome, UPS, gene expression, proteasome inhibition, CRISPR, NIH-3T3, Daoy, Medulloblastoma

## Abstract

Many cellular processes are regulated by proteasome-mediated protein degradation, including regulation of signaling pathways and gene expression. Among the pathways regulated by the ubiquitin–proteasome system is the Hedgehog pathway and its downstream effectors, the Gli transcription factors. Here we provide evidence that proteasomal activity is necessary for maintaining the activation of the Hedgehog pathway, and this crucial event takes place at the level of Gli proteins. We undertook extensive work to demonstrate the specificity of the observed phenomenon by ruling out the involvement of primary cilium, impaired nuclear import, failed dissociation from Sufu, microtubule stabilization, and stabilization of Gli repressor forms. Moreover, we showed that proteasomal-inhibition-mediated Hedgehog pathway downregulation is not restricted to the NIH-3T3 cell line. We demonstrated, using CRISPR/Ca9 mutagenesis, that neither Gli1, Gli2, nor Gli3 are solely responsible for the Hedgehog pathway downregulation upon proteasome inhibitor treatment, and that Cul3 KO renders the same phenotype. Finally, we report two novel E3 ubiquitin ligases, Btbd9 and Kctd3, known Cul3 interactors, as positive Hedgehog pathway regulators. Our data pave the way for a better understanding of the regulation of gene expression and the Hedgehog signaling pathway.

## 1. Introduction

The Hedgehog (Hh) pathway is an important signaling cascade conserved from Drosophila to mammals. It plays a crucial role in embryonic development and tissue regeneration, whereas its defects can lead to developmental disorders and cancer [1]. The effectors of the Hh signaling pathway are three members of the Gli protein family: Gli1, Gli2, and Gli3. When the pathway is inactive, Gli2 and Gli3 are proteasomally processed to their repressor forms (GliR) to suppress target gene expression. On the other hand, when the pathway is in the active state, Gli2 and Gli3 are converted into Gli activators (GliA) in a process which is not fully understood, and induce target gene expression. Gli1, which is transcriptionally upregulated by Hh signaling, acts as a signal amplifier further increasing pathway activity [1].

The Ubiquitin–Proteasome System (UPS) regulates protein homeostasis in the cell. Ubiquitin ligases transfer ubiquitin moieties to target proteins, which in turn targets them for degradation by the multiprotein protease complex known as the proteasome. The UPS plays a key role in the regulation of many signaling pathways, including Hh signaling. All elements of the Hh signaling cascade are regulated by the UPS, starting from the receptor Patched 1 (Ptch1), through Sufu, to Gli transcription factors [2]. In mammals, Ptch1 appears to be regulated by the Nedd4-family HECT domain E3 ligases via its PPXY motifs [3], while Sufu is indirectly regulated by the UPS via Crn and directly via Fbxl17 [4,5].

At the level of Gli proteins, the UPS mediates the formation of GliR and participates in the degradation of GliA, directly affecting pathway activity [1,6]. Two distinct ubiquitin ligase complexes target Gli proteins depending on the pathway state. When the pathway is inactive, multi-site phosphorylation of full length Gli proteins targets them for ubiquitination by the Cul1-Skp1-βTrCP ligase complex. This mediates Gli2 and Gli3 processing by the proteasome, yielding partially truncated GliR forms, which inhibit target gene transcription [6,7]. When the pathway is active, the Cul3-Rbx1-Spop ligase complex targets GliA for degradation [8,9]. In addition, Gli1 is specifically targeted by the E3 ligase Itchy, which does not seem to affect the stability of Gli2/3 [10,11].

It has been long thought that GliA degradation provides a negative feedback loop that allows for the modulation of the pathway activity [12]. Therefore, we hypothesized that proteasome inhibition should increase Hh pathway activity by the stabilization of GliA and preventing the formation of GliR. However, here we provide compelling evidence that proteasome activity is, in fact, necessary for Hh pathway activation and that the observed phenomenon does not depend on the primary cilium, GliR stabilization, GliA nuclear translocation, or Sufu.

## 2. Material and Methods

### 2.1. Molecular Cloning

pLentiguide-Puro or pLentiguide-Neo plasmids carrying sgRNAs were cloned according to the Addgene protocol [13,14]. sgRNAs were designed using the CRISPick [15,16] online tool from Broad Institute. Rbx1 was cloned into pENTR2b using Gibson Assembly method (NEB) and subsequently shuttled in frame with C-terminal V5 tag into pEF5/FRT/V5-DEST (Thermo Fisher, Waltham, MA, USA) using Gateway cloning (LR clonase mix, Thermo Fisher, Waltham, MA, USA) as described before [17].

### 2.2. Cell Culture

HEK293T, NIH-3T3, and Daoy cells were cultured in high glucose DMEM media (Biowest, Nuaillé, France) supplemented with 10% FBS (Eurx, Gdansk, Poland), non-essential amino acids (Thermo Fisher, Waltham, MA, USA), stable glutamine (Biowest), sodium pyruvate (Thermo Fisher, Waltham, MA, USA), and penicillin/streptomycin (Thermo Fisher, Waltham, MA USA). Transient transfections of cells we performed using the JetOptimus reagent (Polyplus, Illkirch-Graffenstaden, France) according to the manufacturer’s protocol. The NIH-3T3 Flp-In (Thermo Fisher, Waltham, MA, USA) cell line was used to generate HA-Gli2(P1-6A), HA-mGli1, HA-TurboID, and HA-Gli2(P1-6A)-TurboID stable cell lines according to the manufacturer protocol. DN Kif3a Gli2(P1-6A) NIH-3T3 were generated as described previously [17]. Stable cell lines were reselected with hygromycin on every other passage to preserve selection pressure. Monoclonal cell lines were generated by the limited dilution method seeding 0.5 cell per well on a 96-well plate. Cell viability was assessed using trypan blue (Thermo Fisher Waltham, MA, USA) exclusion method. Trypan blue was added to cell suspension in a 1:1 ratio. Viable and dead cells were counted using a hematocytometer chamber in duplicates. Proteasome inhibitor treatment with Bortezomib (LC Laboratories, Woburn, MA, USA), MG-132 (VWR, Gdansk, Polska), Carfilzomib (Cayman Chemicals, Ann Arbor, MI, USA), Epoxomicin (Cayman Chemicals, Ann Arbor, MI, USA), and lactacystin (VWR, Gdansk, Polska) was performed in a starving medium (culture medium containing 0.5% FBS) for 24 h unless stated otherwise. Epoxomicin was used in 20 nM concentration unless stated otherwise. Hh pathway was stimulated in NIH-3T3 and Daoy cells with 200 nM SAG, over 24 and 48 h, respectively. Cullin inhibition with MLN4924 (BioVision BioVision, Milpitas, CA, USA) treatment was performed in the starving medium over 24 h.

### 2.3. Luciferase Assay

Luciferase assay was performed as described previously [18]. Briefly, cells were grown to confluence and transfected with plasmids carrying Firefly and Renilla luciferases. After 24 h, cells were treated with indicated proteasome inhibitors in the starving medium. Hh pathway activity was analyzed 48 h post-transfection.

### 2.4. Viral Transduction

To obtain cell lines with stable expression of Cas9 endonuclease, HEK293T were transfected with a mix of helper plasmids pMD2.G, pRSV-Rev, pMDLg/pRRE, and lentiCas9-Blast plasmid from Addgene. Viral vectors were harvested 48 h post-transfection, filtered through a 0.45 µm syringe filter, mixed with polybrene 10 µg/mL, and used to transduce WT NIH-3T3 Flp-In, GFP-reporter, DN Kif3a Gli2(P1-6A), and HA-mGli1 cell lines. Medium was changed 24 h post-transduction to allow for 24 h recovery in the complete medium. Selection with 10 µg/mL blasticidin began 48 h post-transduction. After 7 days of selection, cells were suitable for further experiments.

### 2.5. CRISPR/Cas9 Mutagenesis

Cas9-expressing cells were transduced with lentiviral vectors carrying the sgRNA of interest. To produce lentiviral vectors, HEK293T cells were transfected as described above with the mix of helper plasmids and pLentiguide-Puro or pLentiguide-Neo carrying sgRNA of interest. At 48 h post-transduction, culture medium was changed to selection medium with appropriate antibiotic (puromycin 2 µM, neomycin 500 µg/mL). After 7 days in selection, cells were harvested and analyzed using Western blot, qPCR, or flow cytometry.

### 2.6. Flow Cytometry

NIH-3T3 cells expressing Cas9 and GFP-reporter under the control of Hedgehog responsive element were transduced with lentiviral vectors carrying sgRNA against genes of interest or non-targeting control. After 7 days of selection in a puromycin-containing medium, cells were stimulated with SAG as described above and collected for analysis at LSRFortessa (BD).

### 2.7. SDS-PAGE and Western Blot

NIH-3T3 cells were harvested 24 h post-treatment. Cell lysis was performed in gentle lysis buffer (50 mM Tris at pH 7.4, 1% NP-40 [*v*/*v*], 150 mM NaCl, 0.25% sodium deoxycholate, a protease inhibitor cocktail [1× EDTA-free protease inhibitors, (Sigma, Darmstadt, Germany)], 10 mM NaF, 1 mM Na_3_VO_4_) at 4 °C for 30 min. Cell lysate was purified by centrifugation (20 min, 21,000× *g*) and subsequently denatured in Laemmli buffer containing DTT (65 °C, 30 min). Samples were resolved on a polyacrylamide gel, and the next semi-dry transfer was performed using Trans-Blot Turbo (Bio-Rad, Hercules, CA, USA) on a nitrocellulose membrane. For Western blot, membranes were blocked in 5% non-fat milk in TBST, incubated overnight with primary antibody, washed (4×, 10 min), incubated with secondary antibody (1 h, RT), washed (4×, 10 min), and visualized using Clarity or Clarity Max (Bio-Rad, Hercules, CA, USA) ECL on Amersham ImageQuant 800 (Marlborough, MA, USA). The antibodies used are listed in Table 1.

### 2.8. Nuclear-Cytosolic Fractionation

HA-Gli2(P1-6A) cells were cultured in a 10 cm dish and treated with proteasome inhibitors in starvation medium as indicated. Upon the end of treatment, cells were washed twice with ice-cold PBS and once with ice-cold 10 mM HEPES. Cells were left in fresh ice-cold 10 mM HEPES for 10 min to allow them to swell. After 10 min, the buffer was gently aspirated and cells were scraped in 0.5 mL of modified ice-cold SEAT buffer (10 mM HEPES, 250 mM sucrose, 1× SigmaFast, 1 mM NaF, 1 mM Na_3_VO_4_, and 25 µM MG-132 or 1 µM Bortezomib or 180 nM Epoxomicin). The cell lysate was sheared using a 25 G needle attached to a 1 mL syringe pumping up and down 15 times. Total lysate samples were saved at this point. Next, lysates were centrifuged 900× *g* for 5 min at 4 °C. The supernatant was transferred into a new 1.5 mL tube while the nuclear pellet was resuspended in 1 mL of ice-cold SEAT buffer. Next, both fractions were centrifuged a second time 900× *g* for 5 min at 4 °C. The cytosolic fraction supernatant was transferred to a new 1.5 mL tube and left on ice, while the nuclear fraction supernatant was discarded carefully to avoid disturbing the pellet. The nuclear pellet was resuspended in 550 µL nuclear lysis buffer (1 M HEPES, sucrose 315 mM, 1× SigmaFast, MgCl 1 M, 1 mM NaF, 1 mM Na_3_VO_4_, 0.625 U viscolase) and incubated 10 min on ice. Next, 137.5 µL of 5× cytoplasmic lysis buffer (1× Ripa salts, 5 M NaCl, 10% NP-40, 1 M DTT) was added to both cytoplasmic and nuclear fractions and incubated for 1 h at 4°C with shaking. Next, samples were centrifuged 21,000× *g* at 4 °C for 30 min. Finally, supernatants were collected from both fractions, denatured in Laemmli buffer for 30 min at 65 °C, and subjected to Western blot analysis.

### 2.9. TurboID Proximity Labeling and Mass Spectrometry

HA-Gli2(P1-6A)-TurboID and control HA-TurboID cells were cultured to confluence in a complete culture medium at three 150 mm dishes per sample replicate. Next, cells were serum-starved for 18 h, followed by an 8 h treatment with 20 nM epoxomicin. For the last 4 h of treatment, biotin was added to the medium at a final concentration of 500 µM. Cell lysis was performed on ice by scrapping in 1.5 mL RIPA lysis buffer (50 mM Tris pH 8, 150 mM NaCl, 0.1% SDS, 0.5% sodium deoxycholate, 1% Triton X-100, 1× SigmaFast (Sigma, Darmstadt, Germany), 1 mM PMSF) per dish according to Branon protocol [19] with modifications. Cell lysates were collected in 50 mL falcon tubes and incubated on ice for 30 min for complete lysis. Next, cell lysates were clarified by centrifugation for 30 min at 16,000× *g* at 4 °C. After centrifugation, the supernatant was collected and protein concentration was measured with the BCA assay (Thermo Fisher, Waltham, MA USA) according to manufacturer instructions. Pierce^TM^ streptavidin magnetic beads (Thermo Fisher, Waltham, MA USA) were prewashed in the lysis buffer and 50 µL of beads was added to the lysates per 1 mg of total protein. The beads were incubated overnight at 4 °C. Next, the beads underwent several rounds of washes: twice with 1 mL lysis buffer, once with 1 M KCl 0.1% Triton X-100 (Sigma, Darmstadt, Germany), once briefly with 0.1 M Na_2_CO_3_ 0.1% Triton, once briefly with 2 M urea, 10 mM Tris pH 8, 0.1% Triton, twice with lysis buffer, and lastly twice with 50 mM Tris pH 8 without detergents. Washes were performed in 1 mL volume with rotation for 8 min, except for Na_2_CO_3_ and urea washes where the time was reduced to the minimum to avoid bead aggregation. Moist beads were sent to the IBB MS Lab for analysis.

### 2.10. Proteomic Data Analysis

Raw data were analyzed in Microsoft Excel (Microsoft 365). Non-mouse proteins were filtered out. Normalization factors were calculated using the total peptide count. Proteins that had at least 5 peptides in either DMSO or epoxomicin-treated were considered as strong hits. Proteins that had a ratio to HA-TurboID control of less than 1.3 in both DMSO and epoxomicin-treated samples were considered as non-specifically bound. Proteins were considered enriched in the epoxomicin-treated sample if the relative enrichment ratio was at least 1.5, while proteins were considered depleted if the relative enrichment ratio was equal to or smaller than 0.75.

Proteomic data were analyzed using Cytoscape 3.8.2 to generate and visualize the protein–protein interaction network.

### 2.11. Real-Time qPCR

RNA was isolated using the Extrazol (Blirt, Gdansk, Poland) method according to the manufacturer’s instructions. Reverse transcription was performed using the High-Capacity cDNA Reverse Transcription Kit (Thermo Fisher, Waltham, MA, USA). Real-Time qRT-PCR was performed using the Real-Time 2xHS-PCR Master Mix Sybr B (A&A Biotechnology, Gdansk, Poland) on a LightCycler 480 II qPCR System (Roche, Basel, Switzerland). We used the following custom-designed primers listed in Table 2

### 2.12. Immunostaining and Fluorescent Microscopy

NIH-3T3 cells were cultured on glass coverslips. Cells were fixed in 4% [*w*/*v*] paraformaldehyde in PBS (15 min, RT), then washed (3×, 10 min) in PBS. Subsequently, cells were blocked and permeabilized in 5% [*w*/*v*] donkey serum in 0.2% [*w*/*v*] Triton X-100 in PBS. Cells were incubated with the primary antibodies diluted in a blocking buffer (overnight, 4 °C). Next, coverslips were washed (3× 10 min) with 0.05% [*w*/*v*] Triton X-100 in PBS, followed by incubation with secondary antibodies in the blocking buffer (1 h, RT). Coverslips underwent a second round of washes (3× 10 min) with 0.05% [*w*/*v*] Triton X-100 in PBS and were mounted onto slides using a fluorescent mounting medium with DAPI (ProLong Diamond, Thermo Fisher, Waltham, MA USA). Images were acquired on an inverted Olympus IX-73 (Olympus LS, Tokyo, Japan) fluorescent microscope equipped with a 63× uPLANAPO oil objective and the Photometrics Evolve 512 Delta camera. The antibodies used are listed in Table 1.

For the quantitative analysis of fluorescence intensities, images were acquired with the same settings of exposure time, gain, offset, and illumination. Fluorescent intensities were measured in a semi-supervised manner by a custom ImageJ (Fiji version 2.15.1) script. To calculate the Gli2 nuclear translocation, we calculated the log10 values of the ratios of intensities of the fluorescent signal in the nucleus and the surrounding background in each cell.

### 2.13. Data Analysis

The statistical data analysis was performed using R/RStudio (R 4.4.0, Rstudio 2024.04.1 Build 748). For the processing of the fluorescence images, we used the Fiji/ImageJ suite (version 2.15.1). Statistical significance was calculated using Student’s *t*-test for experiments involving two experimental groups, or ANOVA and Tukey post hoc test for multiple comparisons.

## 3. Results

### 3.1. Proteasome Inhibition Downregulates Hh Signaling

**Figure 1:**
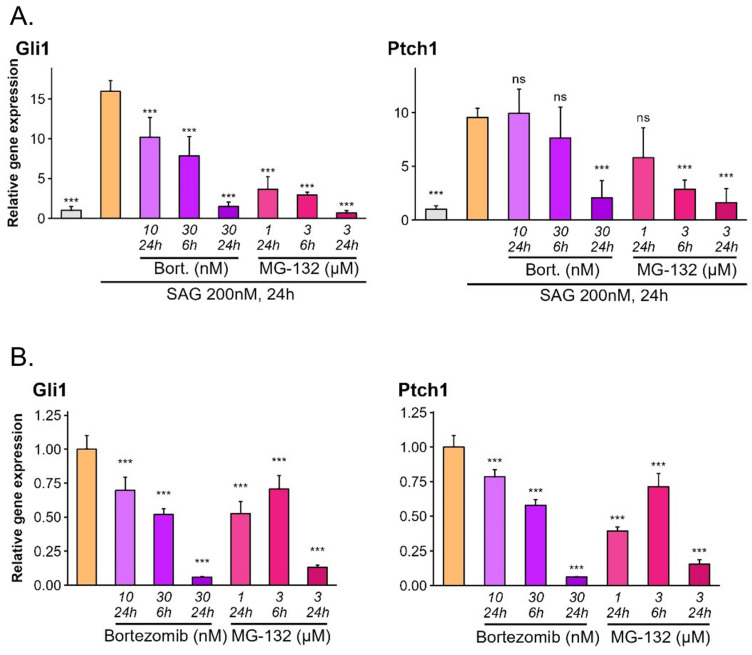

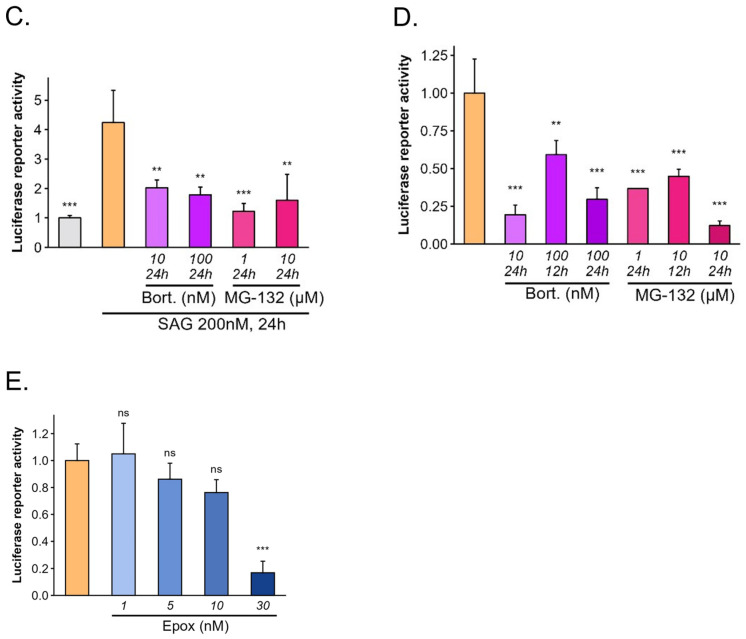
Proteasome inhibition downregulates the Hedgehog pathway. (**A**) mRNA levels of Hh target genes *Gli1* and *Patched* 1 (*Ptch1*) were analyzed by RT-qPCR in NIH-3T3 cells. Cells were serum-starved and treated with proteasome inhibitors as indicated (Bort.—bortezomib) for 24 h. Pathway activity was stimulated with Smo agonist (SAG) 200 nM. Error bars represent the standard deviation (SD) from four replicates. *p*-value: *** *p* < 0.001. (**B**) mRNA levels of Hh target genes *Gli1* and *Ptch1* were analyzed by RT-qPCR in serum-starved NIH-3T3 cells expressing constitutively active Gli2 mutant Gli2(P1-6A) treated with proteasome inhibitors for 6 or 24 h as indicated. Error bars represent SD from four replicates. *p*-value: *** *p* < 0.001. (**C**) Luciferase reporter assay was performed in serum-starved NIH-3T3 cells treated with proteasome inhibitors as indicated (Bort.—bortezomib). Pathway activity was stimulated as in Figure 1A. Error bars represent SD from three replicates except MG-132 10 µM 24 h (n = 2). *p*-value: *** *p* < 0.001, ** *p* < 0.01. (**D**) Luciferase reporter assay was performed in serum-starved NIH-3T3 cells expressing constitutively active Gli2 mutant Gli2(P1-6A) treated with proteasome inhibitors for 12 or 24 h as indicated. Error bars represent SD from three replicates. *p*-value: *** *p* < 0.001, ** *p* < 0.01. (**E**) Luciferase reporter assay was performed in serum-starved NIH-3T3 cells expressing constitutively active Gli2 mutant Gli2(P1-6A) after 24 h treatment with proteasome inhibitor epoxomicin as indicated. Error bars represent SD from three replicates. *p*-value: *** *p* < 0.001. ns: non-significant.

To test how proteasome regulates Hh signaling, we treated NIH-3T3 cells, a Hh model cell line, with increasing doses of two proteasome inhibitors, bortezomib and MG-132, in the presence of SAG, a Smoothened (Smo) agonist. To our surprise, proteasome inhibition caused Hh pathway downregulation measured by the expression of Hh target genes Gli1 and Ptch1 (Figure 1A). We have repeated the same experimental set-up in NIH-3T3 cell line stably expressing Gli2(P1-6A), a constitutively active Gli2 mutant carrying six non-phosphorylatable mutations in a PKA cluster described by us before [20].The same phenomenon was observed, suggesting that the inhibitors block Hh signaling at the level of Gli proteins, i.e., either directly regulating Gli activity or acting through a Gli interaction partner (Figure 1B). We confirmed that observation in cells treated with MG-132, bortezomib, or epoxomicin by an independent method using the Hh reporter luciferase assay, which directly tests the ability of Gli proteins to activate target gene transcription (Figure 1C–E).

To ensure that the proteasome inhibitors cause an accumulation of ubiquitinated proteins in the cell, we performed Western blot experiments on inhibitor-treated cells. Indeed, the ubiquitinated protein abundance increases with proteasome inhibitor treatment (Supplementary Figure S1A,B). Interestingly, all three tested proteasome inhibitors, MG-132, bortezomib, and epoxomicin, were very efficient in the downregulation of Hh pathway activity measured by the level of Gli1 protein, although they varied in the level of induced accumulation of ubiquitin (Supplementary Figure S1A–C). To further verify whether the observed Hh pathway downregulation is not due to the toxicity of proteasome inhibitors we performed an analysis of cell death using the trypan blue exclusion assay, and measured apoptosis by quantifying PARP cleavage. PARP cleavage was not observed for concentrations of the proteasome inhibitors that robustly inhibit the Hedgehog pathway (Supplementary Figure S1D), nor was there an increased number of dead cells in inhibitor-treated samples (Supplementary Figure S1E).

To ensure that the effects we observed are reproducible using different classes of proteasome inhibitors, we tested a broad spectrum of proteasome inhibitors with different molecular modes of action: MG-132, bortezomib, epoxomicin, lactacystin, and carfilzomib. In each case, we observed Hh pathway downregulation (Figure 1A,B,E and Supplementary Figure S1F,G, respectively). Because epoxomicin had given the most consistent dose–response curve in our hands, we decided to use it in subsequent experiments. Finally, to determine if the observed phenomenon is independent of a cell type, we treated the Hh-dependent Daoy human medulloblastoma cell line with a proteasome inhibitor and similarly observed a potent and dose-dependent Hh pathway downregulation (Supplementary Figure S1H).

**Figure 2:**
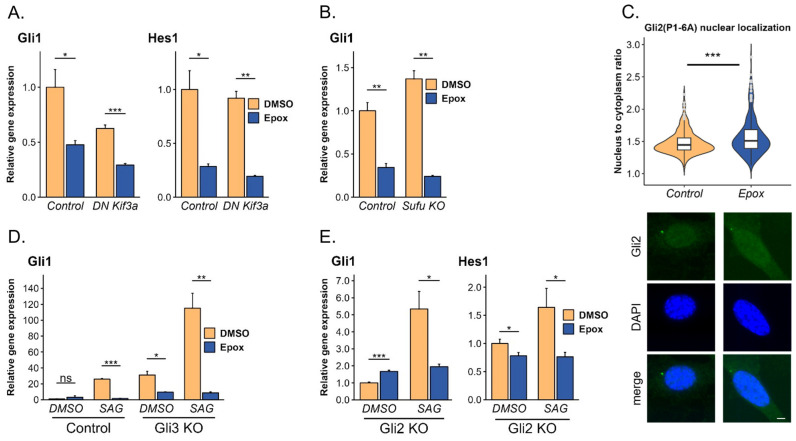

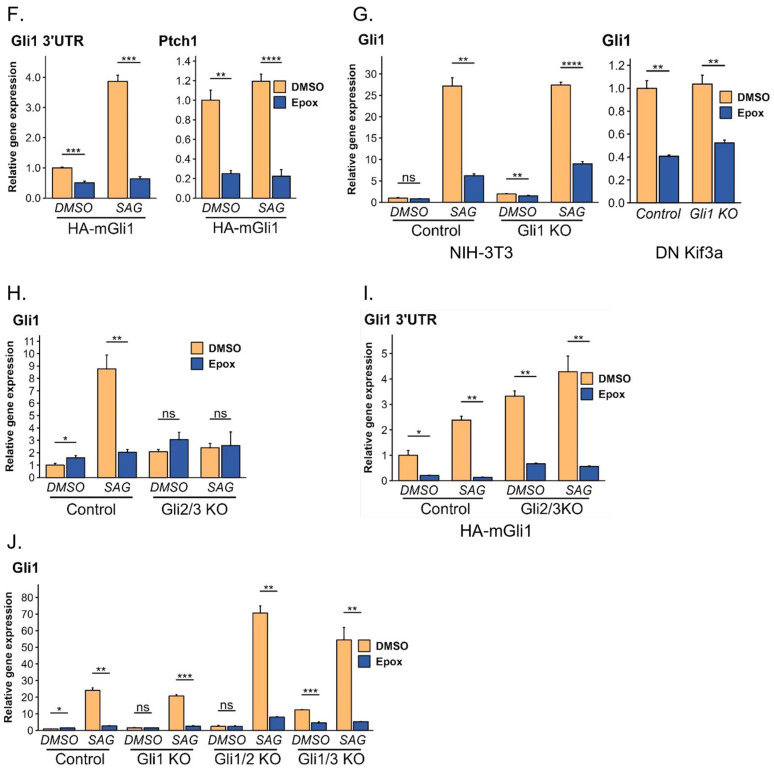
The specificity of the Hedgehog pathway downregulation up proteasome inhibition. (**A**) mRNA levels of Hh target genes *Gli1* and *Hes1* were analyzed after 24 h treatment with 20 nM proteasome inhibitor epoxomicin (Epox) in serum-starved wild-type (WT) or cilia-depleted NIH-3T3 cells expressing dominant negative Kif3a (DN Kif3a) and Gli2(P1-6A) constitutively active mutant. Error bars represent SD from three replicates. *p*-value: *** *p* < 0.001, ** *p* < 0.01, * *p* < 0.05. (**B**) mRNA level of Hh target gene *Gli1* was analyzed in serum-starved WT NIH-3T3 or Sufu knockout (KO) cells as in panel A. Error bars represent SD from three replicates. *p*-value: ** *p* < 0.01. (**C**) Relative nuclear localization of stably expressed Gli2(P1-6A) was analyzed in the NIH-3T3 cell line after 24 h treatment with proteasome inhibitor epoxomicin (Epox). Results are represented as violin plots of the log_10_-transformed ratio of HA-staining fluorescent intensity within the nucleus to the surrounding cytoplasm. Nuclei per condition n > 200. Statistical analysis was performed using Student’s *t*-test (*p*-value: *** *p* < 0.001). Representative images of Gli2(P1-6A) nuclear localization are shown below the graph. DAPI was used as a nuclear stain. Scale bar, 5 µm. (**D**) mRNA level of Hh target gene *Gli1* was analyzed in serum-starved WT NIH-3T3 and Gli3 KO cells as in panel A. Pathway activity was stimulated as in Figure 1A. Error bars represent SD from three replicates except Control NIH-3T3 treated with DMSO and epoxomicin (n = 2). *p*-value: *** *p* < 0.001, ** *p* < 0.01, * *p* < 0.05. (**E**) mRNA levels of Hh target genes *Gli1* and *Hes1* were analyzed in serum-starved NIH-3T3 Gli2 KO cells as in panel A. Pathway activity was stimulated as in Figure 1A. Error bars represent SD from three replicates. *p*-value: *** *p* < 0.001, * *p* < 0.05. (**F**) mRNA levels of Hh target genes *Gli1* and *Ptch1* were analyzed in serum-starved NIH-3T3 stably expressing an exogenous copy of HA-tagged mouse Gli1 (HA-mGli1) as in panel A. Endogenous Gli1 was distinguished using primers against its 3′UTR. Error bars represent SD from three replicates. *p*-value: **** *p* < 0.0001, *** *p* < 0.001, ** *p* < 0.01. (**G**) mRNA level of Hh target gene *Gli1* was analyzed in serum-starved WT NIH-3T3 or DN Kif3a expressing cells upon Gli1 KO as in panel A. Error bars represent SD from three replicates. *p*-value: **** *p* < 0.0001, ** *p* < 0.01. (**H**) mRNA level of Hh target gene *Gli1* was analyzed in serum-starved WT NIH-3T3 or Gli2/Gli3 double KO (Gli2/3 KO) as in panel A. Pathway activity was stimulated as in Figure 1A. Error bars represent SD from three replicates. *p*-value: ** *p* < 0.01, * *p* < 0.05. (**I**) mRNA level of Hh target gene *Gli1* was analyzed in serum-starved HA-mGli1 NIH-3T3 or Gli2/3 KO cells as in panel A. Pathway activity was stimulated as in Figure 1A. Endogenous Gli1 was distinguished as in panel F. Error bars represent SD from three replicates. *p*-value: ** *p* < 0.01, * *p* < 0.05. (**J**) mRNA levels of Gli1 were analyzed in WT NIH-3T3 cell line upon Gli1 KO, Gli1/2 double KO, and Gli1/3 double KO as in panel A. Error bars represent SD from three replicates. *p*-value: *** *p* < 0.001, ** *p* < 0.01, * *p* < 0.05. ns: non-significant.

The fact that proteasome inhibitors block the transcriptional activation triggered by the constitutively active Gli2(P1-6A) mutant suggests that the proteasome regulates Gli activators independently of upstream elements of the pathway, such as Ptch1 and Smo. Because the activation of Gli proteins is a multi-step process, we wanted to establish at which step Gli protein activation is regulated by the proteasome. Numerous studies, including our own recently published [17], demonstrated that activation of Gli proteins is inextricably linked with their translocation to the primary cilium [21,22,23,24,25]. Interference in this process [22,23,24,25] or disruption of primary cilium formation and function [21,26,27,28] were shown to impair the Hh signaling. To determine if the observed phenomenon is the result of abnormal transport of Gli proteins or their processing at the primary cilium [29], we measured the ability of epoxomicin to block constitutively active Gli2(P1-6A) mutant in cells expressing the dominant negative (DN) Kif3a protein, which disrupts cilia formation [17]. We showed that proteasome inhibition can downregulate the Hh pathway even in the absence of the primary cilium (Figure 2A), suggesting that the presence and function of the primary cilium is not the limiting factor mediating Hh pathway downregulation by proteasome inhibitors.

In addition to the regulation of ciliary transport and processing, another potential mechanism that might contribute to the proteasome inhibition effect on Gli function might be the stabilization of or stronger association with Sufu, a known negative regulator of Gli proteins. To determine if the proteasome inhibition effect on Gli function is mediated through Sufu, we treated NIH-3T3 Sufu knock-out (KO) [30] cells with epoxomicin. We observed potent Hh pathway downregulation in cells deprived of Sufu, ruling out the involvement of this protein in the effects of proteasome inhibitors on Gli proteins (Figure 2B).

It was demonstrated, in the context of ovarian cancer, that proteasome inhibition can downregulate Hh pathway activity via tubulin acetylation [31]. We decided to test this mechanism in our experimental setup. We treated NIH-3T3 cells expressing constitutively active Gli2(P1-6A) mutant with bortezomib, or Taxol as a positive control, and did not observe increased tubulin acetylation in bortezomib-treated cells (Supplementary Figure S2A). Additionally, Taxol treatment had no impact on the Hh target genes expression, suggesting that microtubule stabilization does not affect Hh signaling in our experimental setup (Supplementary Figure S2B).

To drive target gene expression activated Gli proteins must translocate from the primary cilium to the nucleus [30,32,33]. We wanted to investigate whether proteasome inhibition can negatively affect the nuclear import of active Gli forms. To that end, we treated NIH-3T3 Gli2(P1-6A) cell line expressing constitutively active Gli2 mutant and measured its nuclear localization upon proteasomal inhibitor treatment. We show that proteasome inhibition has no negative impact on Gli2(P1-6A) nuclear localization. To the contrary, we observed statistically significantly stronger Gli2(P1-6A) nuclear localization in cells treated with epoxomicin (Figure 2C) showing no impairment of Gli2 nuclear import. To further support this observation, we performed a nuclear-cytoplasmic fractionation and again we did not observe a decrease in the level of nuclear Gli2(P1-6A) upon proteasome inhibition (Supplementary Figure S2C,D).

### 3.2. All Three Gli Proteins Are Sensitive to Proteasome Inhibitors

The evidence collected thus far indicates that proteasome inhibition affects the Hh pathway at the level of Gli proteins. To elucidate which Gli protein mediates Hh pathway downregulation we decided to generate cell lines harboring a single KO in each of the three Gli proteins. Although both Gli2 and Gli3 act as transcriptional activators, Gli3 can be proteasomally truncated to its repressor form (Gli3R) [6,34] to inhibit Hh pathway activity. We hypothesized that the attenuation of the Hh pathway activity mediated by proteasome inhibition could be attributed to the selective stabilization and accumulation of Gli3R. However, Gli3 KO cells treated with epoxomicin exhibited potent Hh pathway downregulation (Figure 2D and Supplementary Figure S2E, respectively), suggesting that Gli3, including Gli3R, is not solely responsible for the Hh pathway inhibition. Moreover, when we treated NIH-3T3 cells with epoxomicin we did not observe stabilization of Gli3R. On the contrary, the Gli3R level decreased (Supplementary Figure S2F), suggesting that the effect of epoxomicin on preventing Gli3R formation from full-length Gli3 is stronger than that on inhibiting Gli3R degradation. Next, we reasoned that Gli2, as a major Hh pathway activator, could be directly regulated by the UPS. We treated Gli2 KO with epoxomicin and observed strong Hh pathway downregulation (Figure 2E and Supplementary Figure S2G, respectively), suggesting that similarly to Gli3, Gli2 is not solely responsible for the Hh pathway downregulation mediated by proteasome inhibition. Finally, we verified the role of Gli1, which in non-stimulated cells is expressed at very low levels, and upon pathway activation is transcriptionally upregulated and acts as a signal amplifier providing a positive feedback loop. To establish whether stable expression of exogenous Gli1 could abolish the effect of proteasome inhibition on the Hh pathway, we treated NIH-3T3 cells stably expressing exogenous copy of mouse Gli1 (HA-mGli1) with a proteasome inhibitor. We observed strong Hh pathway downregulation (Figure 2F), suggesting that Gli1 is susceptible to proteasome-mediated Hh pathway inhibition. To verify if Gli1 is the sole factor responsible for the observed phenomenon, we generated Gli1 KO cells (Supplementary Figure S2H) and treated them with epoxomicin. We observed strong downregulation of pathway activation in both normal and cilia-less cells that lack a functional copy of Gli1 (Figure 2G), suggesting that neither Gli1 nor Gli2 or Gli3 is solely responsible for the pathway downregulation.

Although none of the Gli proteins solely mediates Hh pathway downregulation, a combination of two or even all three proteins may be involved in the process. Gli2 and Gli3 are the primary effectors of the Hh pathway. Therefore, we hypothesized that they may redundantly mediate the effects of proteasome inhibition on the Hh pathway. Although the double Gli2/3 KO renders NIH-3T3 cells insensitive to Hh pathway activation (Figure 2H and Supplementary Figure S2I, respectively), we employed two alternative models to verify our hypothesis. In the first model, we generated Gli2/3 double KO in the WT NIH-3T3 cell line and performed a rescue experiment with the constitutively active Gli2(P1-6A). In the second one, we generated Gli2/3 double KO in HA-mGli1 NIH-3T3 cells. In both models, when treated with the proteasome inhibitor, the cells exhibited strong Hh pathway downregulation (Supplementary Figure S2J and Figure 2I, respectively), suggesting that both Gli1 and Gli2(P1-6A) can mediate proteasome inhibition-induced Hh pathway downregulation. Lastly, to assess whether Gli1 in combination with Gli2 or Gli3 is responsible for the observed phenomenon, we generated Gli1/2 and Gli1/3 double KO in the NIH-3T3 cell line (Supplementary Figure S2K). Both double KO cell lines were treated with the proteasomal inhibitor and showed strong Hh pathway downregulation (Figure 2J), suggesting that all three Gli proteins can mediate the Hh pathway downregulation upon proteasome inhibition.

### 3.3. Interactome of Gli2 Changes upon Proteasome Inhibition

The activity of Gli transcription factors is regulated by their interaction with a variety of transcription co-activators and co-repressors. To verify whether proteasome inhibition results in the specific change in the interaction of Gli proteins with one or more of its binding partners, we performed the proximity labeling BioID assay [19] coupled with mass spectrometry. We generated a Gli2(P1-6A)-TurboID NIH-3T3 cell line and treated the cells with epoxomicin. The analysis of mass spectrometry data indeed revealed alterations in the Gli2 interactome in cells treated with the proteasome inhibitor (Supplementary Figure S3A). We identified several proteins that were either enriched (see Table S1) or depleted (see Table S2) in the Gli2 interactome in epoxomicin-treated cells. Amongst the “enriched” subset we observed several proteins involved in the regulation of gene expression, including Morc4, Sin3a, Med12, and Nipbl. Additionally, three myosin heavy chain subunits, Myh1, Myh4, and Myh8, were strongly enriched in the Gli2 interactome of epoxomicin-treated cells (see Table S3). We hypothesized that one or more of the proteins that interacted more strongly with Gli2 in epoxomicin-treated cells (the “enriched” dataset) would be required for the effects of epoxomicin on Gli1 expression. We tested seven of the genes from the “enriched” dataset using CRISPR/Cas9 mutagenesis. However, the mutation of neither of the genes was able to prevent epoxomicin-mediated Hh pathway downregulation (Supplementary Figure S3B).

### 3.4. Identification of Ubiquitin Ligase Responsible for Proteasome-Dependent Activation of Gli Proteins

**Figure 3:**
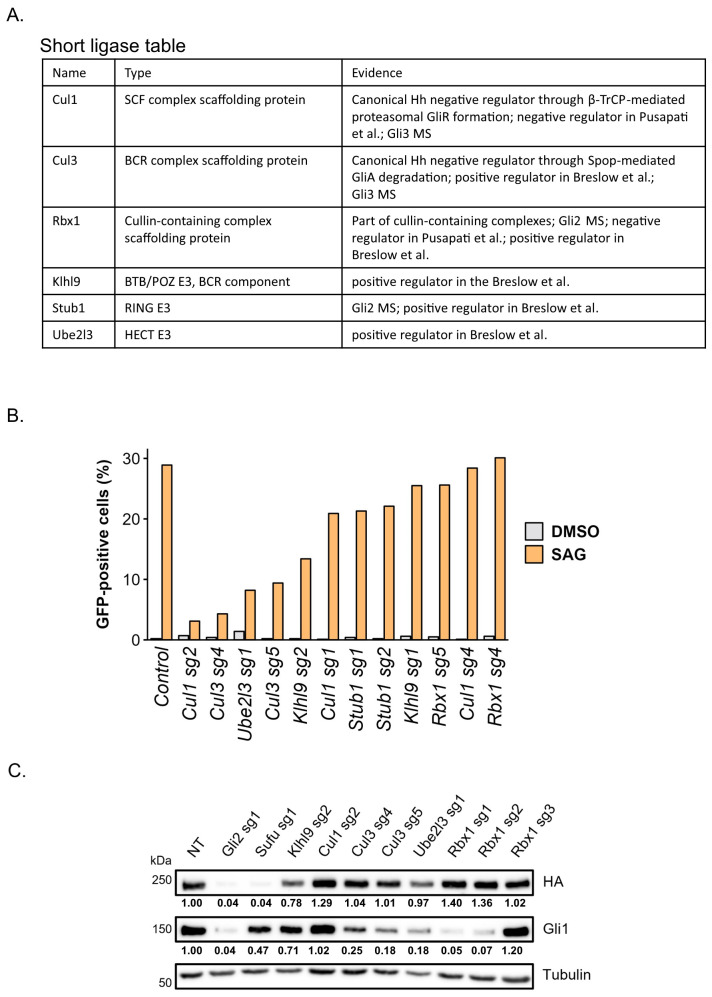
E3 ubiquitin ligase screening. (**A**) Table of ubiquitin ligases or ubiquitin ligase complex components selected for CRISPR/Cas9 mediated screen. (**B**) CRISPR/Cas9 screen in serum-starved NIH-3T3 reporter cell line with GFP under Gli-responsive promoter. The effect of gene KO on the Hh pathway was analyzed by flow cytometry as a fraction of GFP-positive cells. Two sgRNAs per gene were used. Pathway activity was stimulated as in Figure 1A. (**C**) CRISPR/Cas9 screen in serum-starved NIH-3T3 cells expressing constitutively active HA-tagged Gli2(P1-6A) mutant. Protein levels of HA-Gli2(P1-6A) and Gli1 were analyzed upon KO of indicated genes. Non-targeting sgRNA (NT) and sgRNAs against Gli2 and Sufu were used as controls. Tubulin was used as a loading control. Relative increase/decrease of Gli2 and Gli1 protein levels normalized to tubulin and compared to NT samples is shown below the respective bands [17,36,37].

Protein degradation by the proteasome depends on their prior ubiquitination event mediated by a ubiquitin ligase complex. Our discovery that inhibition of the proteasome causes a reduction in Gli protein activity suggests that the Ubiquitin-Proteasome System plays a dual role in Hh signaling. On the one hand, ubiquitination of Gli3, and to some extent Gli2, by the Cul1-β-TrCP complex causes their proteasomal conversion into repressor forms (Gli2/3R) [6,35]. On the other hand, our results suggest that there are likely ubiquitin ligases whose role in the pathway is the opposite and that they are necessary for the function of Gli activators (Gli2/3A). Loss of function of these ligases should prevent proteasomal degradation of Gli proteins, mimicking proteasome inhibition. In order to identify a ubiquitin ligase complex involved in the positive regulation of the Hh pathway, we combined data from large-scale co-IP/MS and CRISPR/Cas9 screens to create a list of potential candidate ligases for this function. We have chosen ubiquitin ligases that were either Gli2 or Gli3 interactors or were identified as positive Hedgehog pathway regulators in functional Hedgehog CRISPR screens (Figure 3A) [36,37]. We then performed a small-scale CRISPR/Cas9 functional screen in an NIH-3T3 reporter cell line expressing GFP under a Gli-responsive promoter (Figure 3B). After transduction with sgRNAs, the cells were treated with SAG, and Hh pathway activity was measured using GFP flow cytometry. As a result of the screening, we identified Cul1, Cul3, Klhl9, and Ube2l3 as promising candidates and we confirmed that the drop in their activity is not due to the loss of primary cilia (Supplementary Figure S4). For the second round of screening, we chose cells expressing constitutively active Gli2(P1-6A) to validate results from the first screen. Additionally, we expanded the screen to include Rbx1, an essential interactor of cullin-family ligases, and decided to use different sgRNAs than in the first screen to ensure result specificity (Supplementary Figure S5A). We found that Klhl9 KO had a marginal effect on the Hh signaling, while Ube2l3, Cul3, and Rbx1 are required for the Hh pathway activity measured as the level of Gli1 protein (Figure 3C). Ube2l3 is an E2 ubiquitin-conjugating enzyme, and as such participates in a variety of ubiquitination events [38,39,40,41], thus we decided not to pursue it further as a potential target.

**Figure 4:**
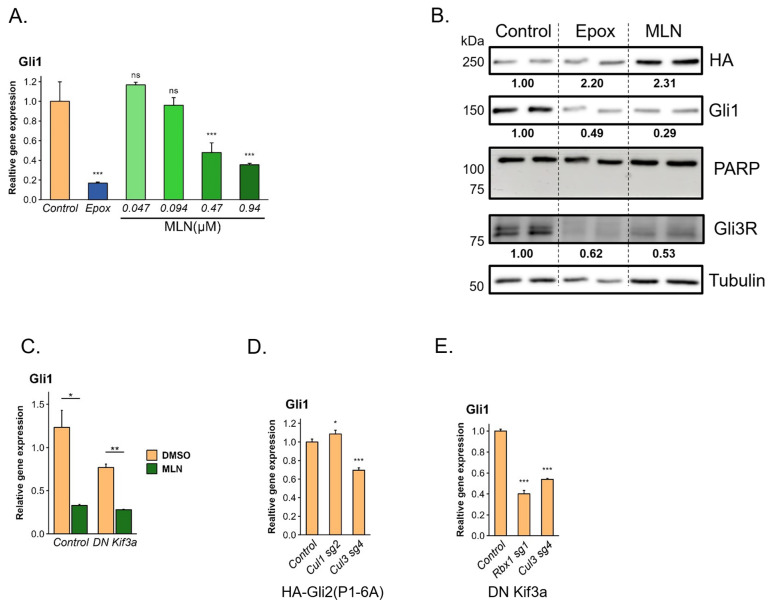
Inhibition of the cullin family of E3 ubiquitin ligases downregulates Hh pathway activity. (**A**) mRNA level of Hh target gene *Gli1* was analyzed in serum-starved NIH-3T3 expressing constitutively active Gli2(P1-6A) mutant after 24 h treatment with cullin inhibitor MLN4924 (MLN) as indicated. Error bars represent SD from three replicates. Error bars represent SD from three replicates. *p*-value: *** *p* < 0.001. (**B**) Protein levels of HA-tagged Gli2(P1-6A), Gli1, Gli3R, and PARP/cleaved PARP were analyzed in serum-starved NIH-3T3 expressing constitutively active Gli2(P1-6A) mutant after 24 h treatment with 0.94 µM cullin inhibitor (MLN) or 20 nM proteasome inhibitor epoxomicin (Epox). Tubulin was used as a loading control. Gli2 and Gli1 mean protein levels normalized to tubulin and relative to non-treated control are shown below the respective bands. (**C**) mRNA level of Hh target gene *Gli1* was analyzed in serum-starved WT or cilia-less NIH-3T3 cells expressing DN Kif3a mutant after 24 h treatment with 0.94 µM MLN. Error bars represent SD from three replicates. *p*-value: ** *p* < 0.01, * *p* < 0.05. (**D**) mRNA level of Hh target gene *Gli1* was analyzed in serum-starved NIH-3T3 expressing constitutively active Gli2(P1-6A) mutant upon KO of *Cul1* or *Cul3*. Non-targeting sgRNA was used as a control. Error bars represent SD from three replicates. *p*-value: *** *p* < 0.001, * *p* < 0.05. (**E**) mRNA level of Hh target gene *Gli1* was analyzed in serum-starved cilia-less DN Kif3a NIH-3T3 cells expressing constitutively active Gli2(P1-6A) mutant upon KO of *Cul3* and *Rbx1* genes. Non-targeting sgRNA was used as a control. *p*-value: *** *p* < 0.001. ns: non-significant.

Consistent with the effect of the knockout of the cullin Cul3 and its interaction partner Rbx1, we observed a direct dose-dependent Hh pathway downregulation by the cullin inhibitor MLN4924, similar to the effects of proteasome inhibitor treatment (Figure 4A). To rule out a non-specific effect of MLN4924 on cell viability, we checked PARP cleavage in cells treated with the drug but did not observe any cleaved PARP for any of the doses used (Figure 4B). The effect of MLN4924 was also apparent in cilia-less cells expressing Gli2(P1-6A), suggesting a cilium-independent effect on Gli proteins (Figure 4C). We analyzed the effects of the knockout of Cul3 and Cul1 KO in Gli2(P1-6A) cell line by qPCR and for the two cullin members, only Cul3 KO resulted in Hh pathway downregulation (Figure 4D, Supplementary Figure S5B). We further strengthened the notion of the involvement of Cul3 in the activation of Hh pathway through the mutation of Cul3 and its interacting partner Rbx1 in cilia-less cells. We observed a strong Hh pathway downregulation upon KO of Cul3 and Rbx1 in DN Kif3a-expressing NIH-3T3 cells (Figure 4E), suggesting that the effect on the Hh pathway is independent of the primary cilium.

**Figure 5:**
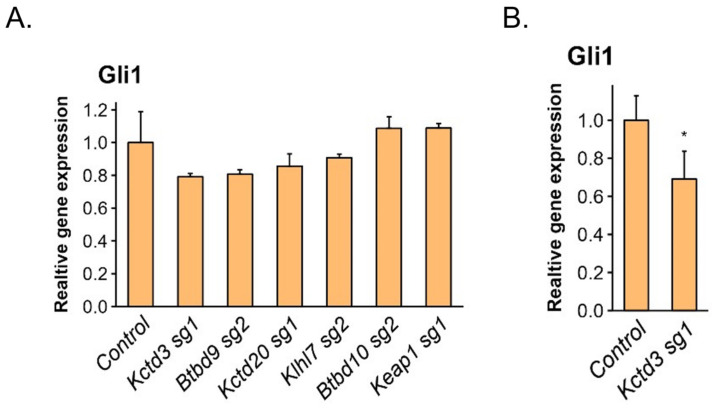

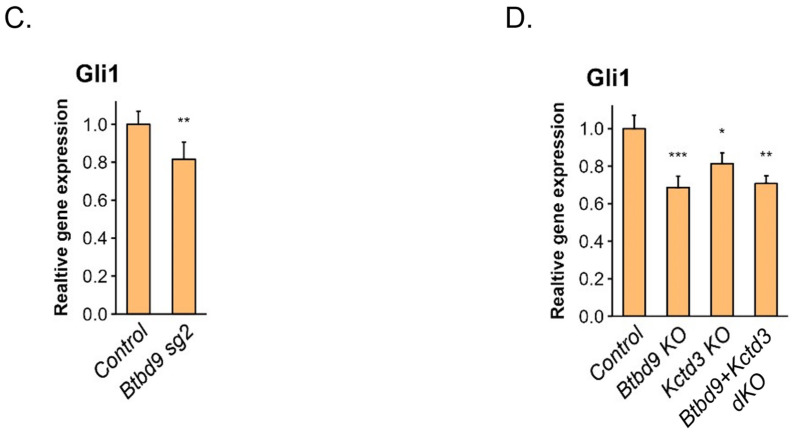
CRISPR/Cas9 screening of Cul3 interacting partners. (**A**) mRNA level of Hh target gene *Gli1* was analyzed in serum-starved NIH-3T3 expressing constitutively active Gli2(P1-6A) mutant upon KO of indicated genes. Non-targeting sgRNA was used as a control. Error bars represent SD from three replicates. (**B**) mRNA level of Hh target gene *Gli1* was analyzed in serum-starved NIH-3T3 expressing constitutively active Gli2(P1-6A) mutant upon KO of the *Kctd3* gene. Non-targeting sgRNA was used as a control. Error bars represent SD from four replicates. *p*-value: * *p* < 0.05. (**C**) mRNA level of Hh target gene *Gli1* was analyzed in serum-starved WT NIH-3T3 upon KO of the *Btbd9* gene. Pathway activity was stimulated as in Figure 1A. Non-targeting sgRNA was used as a control. Error bars represent SD from Control (n = 4) and Btbd9 sg2 (n = 6). *p*-value: ** *p* < 0.01. (**D**) mRNA level of Hh target gene *Gli1* was analyzed in serum-starved cilia-less DN Kif3a NIH-3T3 cells expressing constitutively active Gli2(P1-6A) mutant upon single or double KO of *Btbd9* and *Kctd3* genes. Non-targeting sgRNA was used as a control. Error bars represent SD from three replicates. *p*-value: *** *p* < 0.001, ** *p* < 0.01, * *p* < 0.05.

Cullins are scaffolding proteins, which bind various adaptor proteins, ensuring their specificity towards different substrates. To identify Cul3 interactors that are important for the function of Cul3 in Gli protein regulation, we combined Gli3 and Gli2 interactome data, as well as information on previously identified Gli-interacting E3 ligases reported in the literature. We analyzed six Cul3 interacting partners and potential regulators of Hh signaling (Figure 5A) and identified Btbd9 and Kctd3 as E3 ligases that act as novel positive regulators of Gli activity (Figure 5B,C and Supplementary Figure S5C,D, respectively). To determine if the effect of the knockout of Btbd9 and Kctd3 is a result of the abnormal function of primary cilia we generated Btbd9 and Kctd3 KO in DN Kif3a-expressing cilia-less cells. Finally, we verified the effect of Btbd9 and Kctd3 KO in cilia-less cells individually and in combination. We confirmed that the individual KO of both Btbd9 and Kctd3 significantly downregulated the Hh pathway activity, but no synergistic effect was observed in the double KO (Figure 5D).

## 4. Discussion

The need to better understand the regulation of the Hedgehog pathway is highlighted by the broad spectrum of malignancies driven by its abnormal activity, including medulloblastoma, basal cell carcinoma, gastric cancer, and others [1]. Although the Hh pathway has been intensively studied over the years, many of its aspects remain unclear. In particular, we only have fragmentary knowledge of the modulation of Hh signaling by the UPS [42], whose role is well-documented in other signaling cascades, such as NF-κB [43] and WNT [44].

The involvement of the UPS in the regulation of the Hedgehog pathway has been studied at all levels of the signaling cascade starting from the receptor Patched, through Sufu, to the pathway effectors, i.e., the Gli transcription factors [2]. When the Hh pathway is inactive, Gli proteins Gli2 and Gli3 in mammals, or their homolog Ci in Drosophila, are partially degraded by the proteasome to their repressor forms [6,34,35]. On the other hand, when the pathway is activated, Gli2/3 proteins are targeted for proteasomal degradation by the Cul3-Spop ubiquitin ligase complex [42,45,46]. Interestingly, the degradation of IκB, β-catenin, and Gli2/3 processing to GliR occurs with the involvement of the E3 β-TrCP E3 ubiquitin ligase.

In the context of the Hh pathway, we were expecting that proteasome inhibitor treatment would likely increase the pathway activity due to the prevention of GliR formation by Cul1-β-TrCP and GliA degradation by Cul3-Spop. To our surprise, the treatment of the NIH-3T3 cells with various proteasomal inhibitors, MG-132, bortezomib, epoxomicin, lactacystin, and carfilzomib, had a strong inhibitory effect on the pathway activity. The fact that proteasome inhibitors block the activity of Gli2(P1-6A), a Gli2 constitutively active mutant, suggests that the effect is directly mediated through Gli proteins rather than upstream.

We initially suspected that the effect of proteasome inhibition on Hh signaling might be due to its effect on the formation of primary cilia, organelles that are essential for full Gli protein activation [1,47]. It has been shown that proteasomal degradation of the Aurora-A kinase activator trichoplein by the Cul3-Kctd17 complex is necessary for the initiation of ciliogenesis [29]. However, we showed that the lack of functional primary cilia did not prevent the effect of proteasome inhibition on constitutively active Gli function, suggesting that the observed phenomenon is not an effect of aberrant ciliogenesis.

We also wondered if proteasome inhibitors might affect Gli proteins via Sufu, a key Hh pathway negative regulator that was shown to act independently of the primary cilium [48] which is also known to be regulated by the UPS [49]. Proteasome-mediated Sufu degradation, induced by the binding of the Shh ligand to the Patched receptor, promotes Sufu degradation and in turn activates Gli2/3 [49]. In mammalian cells, an aberrant decrease in Sufu protein level leads to Hh pathway activation regardless of upstream events [50]. Two E3 ligases were shown to regulate Sufu protein levels. In Drosophila, HIB/Spop indirectly regulates Sufu through the spliceosome member Crn [4]. On the other hand, a direct ubiquitination of Sufu is mediated by Fbxl17, which targets it for proteasomal degradation in the nucleus [5]. We hypothesized that Sufu stabilization might underlie the Hh gene expression downregulation by proteasome inhibitors. We addressed that possibility using Sufu KO cells [30] and demonstrated that even in the absence of Sufu proteasome inhibition can strongly downregulate the Hh pathway, suggesting that Sufu is not critical for the effects of proteasome inhibitors on Hh signaling. Additionally, the knockout of Cul1, a crucial interaction partner of Fbxl17 [5], did not affect Hh target gene expression triggered by Gli2(P1-6A), suggesting that Gli2(P1-6A), unlike Sufu, is not affected by the Cul1-Fbxl17 axis.

From among the three Gli proteins, Gli2 and Gli3 are bifunctional and act as repressors or activators, while Gli1 acts solely as an activator and signal amplifier [1]. As the Hh pathway downregulation mediated by proteasome inhibition could affect any of the Gli proteins, we analyzed the effect of proteasome inhibition in Gli knockouts. None of the Gli single KOs abolished the effect of the proteasome inhibitor treatment, suggesting redundancy. As double KO of Gli2 and Gli3 renders NIH-3T3 cells non-responsive to Hh pathway activation, we used two approaches to determine the possible redundancy between Gli2 and Gli3. In the first approach, we performed a rescue experiment with constitutively active Gli2(P1-6A) mutant, while in the second approach, we performed double Gli2/3 KO in NIH-3T3 cell line stably expressing an additional copy of Gli1. In either case, we observed potent Hh pathway downregulation mediated by proteasome inhibition suggesting that all three Gli proteins are involved in the process.

The UPS consists of three enzymes, E1, E2, and E3, which sequentially mediate the transfer of a ubiquitin moiety to the target protein, and the 26S proteasome, which recognizes and degrades ubiquitinated proteins [51]. Although in humans there are only two E1 ubiquitin-activating enzymes [52] and less than 50 E2 ubiquitin-conjugating enzymes [53], the specificity of each reaction and the proper function of the system is conferred by over 600 E3 ubiquitin ligases [54], vastly increasing the complexity of the identification of ligases responsible for the ubiquitination of specific targets.

Screening for potential E3 ligases that regulate Gli proteins narrowed down the list of candidates to the cullin-RING family. Indeed, Gli protein activity was blocked by the cullin inhibitor MLN4924 and by the knockout of Rbx1, a component of all cullin complexes apart from Cul5 [55,56]. From among the seven members of the cullin family, Cul1 and Cul3 had previously been established in the regulation of Hh signaling. In our experimental setup, only the knockout of Cul3 resulted in the downregulation of Hh signaling in both SAG-stimulated and Gli2(P1-6A)-expressing cells, suggesting a direct effect on active Gli proteins. This result prompted us to screen Cul3-interacting ubiquitin ligases, among which we identified Btbd9 and Kctd3 as positive Hh pathway regulators both in wild-type and cilium-depleted cells. However, even the KO of both of these ligases resulted in weaker Hh pathway downregulation compared to the treatment with proteasome and cullin inhibitors, and to the KO of Cul3 and Rbx1. This suggests that there may be additional Cul3/Rbx1 interaction partners that are involved in the direct regulation of Gli proteins.

Both Btbd9 and Kctd3 belong to the BTB domain-containing protein superfamily, a large family of heterogeneous proteins with diverse roles and functions, many of which form E3 complexes with Cul3 and Rbx1. Several of the BTB domain proteins have been reported to regulate the Hh signaling pathway including Spop, and members of the Kctd family. Among them are Kctd11, Kctd21, and Kctd6, which indirectly regulate Gli1 and Gli2 deacetylation through degradation of Hdac1 [57,58], Kctd1 and Kctd15, involved in the regulation of Kctd11 and Kctd21 [59,60], and the previously described Kctd17, involved in the initiation of ciliogenesis [29].

Despite the broad involvement of BTB-domain containing E3 ligases in Hh pathway regulation, neither Btbd9 nor Kctd3 had been previously implicated in Gli protein modulation. Btbd9 has mostly been studied in the context of its role in Restless Legs Syndrome [61,62,63] and the regulation of sleep by its substrates [64]. As for Kctd3, little is known about its role in mammalian physiology, with the few reports showing its influence on milking speed in cattle [65], carrying over reproductive traits in pigs [66], and neurogenetic disorders in humans [67,68] including epileptic encephalopathy [69].

Our finding that inhibition of the proteasome, as well that of as BTB-Cul3-RING E3 ligases, blocks the transcriptional activity of Gli proteins despite increasing their protein levels appears paradoxical. However, such counterintuitive dependency of transcription on the UPS has previously been reported for a few transcriptional activators. Two main mechanisms have been described: either an inhibitory interaction partner of the transcription factor is degraded via the UPS or the recycling of the transcription factor itself by proteasomal degradation is required for the maintenance of its activity. The latter model is supported by the fact that in many transcription factors, the activation domain coincides with the degradation domain [70], and some highly active transcription factors are characterized by low expression levels and high turnover rates [71].

A well-studied example of a transcription factor that is activated by the UPS is the yeast protein Gal4. While initially it was surmised that the direct recycling of Gal4 was responsible for the positive effect of the UPS on its transcriptional activity [71,72], later it was shown that it is the degradation of Gal4 inhibitor Gal80 that plays a pivotal role in this process [73]. On the other hand, for the human transcription factor MYC, the role of its direct degradation via the UPS in the maintenance of its transcriptional activity was demonstrated directly using ubiquitination-resistant lysine-less mutants [74]. Similarly, for the Erα, the continued transcriptional activity requires its UPS-mediated turnover [75]. For HIF1α, another transcription factor that is negatively regulated by proteasome inhibitors, the mechanism of transcriptional activity attenuation is unclear [76].

Our study does not rule out either mechanism of inhibition by proteasome blockers. Gli transcription factor activity may be directly regulated by the proteasome either via its clearance from the chromatin, as it is in the case of MYC or Erα [75], or indirectly by stabilization of an unknown Gli inhibitor, as for Gal4. Indeed, several nuclear interactors that could potentially affect Gli protein transcriptional activity were enriched in the Gli2 interactome in epoxomicin-treated cells, such as Med12 [77] and Sin3a [78]. Additionally, Gli2 interacts with a few proteins exclusively in epoxomicin-treated cells: myosin heavy chain subunits Myh1, Myh4, and Myh8 as well as Actin α1. Although these proteins have been mostly implicated in skeletal muscle functions, there is some evidence linking them with Hh signaling. Myh4 was identified as a weak Hh negative regulator in the CRISPR/Cas9 screen performed by Pusapati et al. [37]. Myh4 is associated with Retinitis pigmentosa 67, a subtype of known ciliopathy associated with ciliary kinase Nek2 [79] while mutations in Myh8 were found in a variant of Carney Complex [80], a hereditary disease associated with mutations in Prkar2b, a regulatory subunit of protein kinase A, a key regulator of Hh signaling [25,81]. Nevertheless, when we tested these interactors in a small-scale CRISPR/Cas9 mutagenesis screen, neither proved to be critical for the effect of proteasome inhibitors on Hh signaling. In the future, a more comprehensive screen of Gli interactors may yield more insight as to the precise mechanism of the impact of UPS inhibition on Gli function.

Abnormal Hh pathway activation is a driving force in various types of human cancer [1], but their treatment still presents a challenge. Despite many years of research, only two Hh inhibitors, vismodegib and sonidegib, have been approved for treatment in the US and the EU. Both chemicals target aberrant Hh pathway activity upstream of Sufu and Gli proteins, and their use is limited to a narrow spectrum of malignancies. Although discovered in 2007 as the first Gli-specific inhibitor [82], GANT61 did not enter clinical trials, likely due to its unfavorable pharmacokinetic profile [83]. More recently, new Gli-specific inhibitors, a natural isoflavone Glabrescione B [84,85] and quinoline derivative JC19 [86], were reported; however, the research on them is at the early stage. In this context, proteasome inhibition, which strongly downregulates the Hh pathway emerges as a promising therapeutic approach in Hh-driven malignancies. Proteasome inhibitors such as bortezomib and carfilzomib possess yet another advantage over specific Gli inhibitors, as they are FDA-approved drugs in the treatment of multiple myeloma, which greatly simplifies the process for new therapy approval. In our study, we demonstrated as a proof of concept that proteasome inhibition strongly downregulates Hh pathway activity in Daoy cells, a model cell line for human medulloblastoma, paving the way for future translational research.

## 5. Conclusions

In summary, we report a novel mechanism of Hedgehog pathway regulation by the Ubiquitin-Proteasome System. We demonstrated that the proteasome activity is necessary to sustain Gli transcription factor activity and that proteasome-mediated regulation occurs at the level of Gli proteins. We present Cul3/Rbx1 as the most likely E3 scaffold which participates in the positive regulation of Hh signaling by the UPS. Finally, we identify two ubiquitin ligases, Btbd9 and Kctd3, as novel positive regulators of the Hh pathway and potential therapeutic targets for Hh-related malignancies.

## Figures and Tables

**Table 1:** The list of antibodies used in all experiments.

Name	Manufacturer	Catalog Number	Application (Dilution)
Primary antibodies			
Anti-Gli2	R&D Systems (Minnneapolis, MN, USA)	AF3635	WB (1:1000)
Anti-Gli1	Cell Signaling Technologies (Danvers, MA, USA)	2643	WB (1:1000)
Anti-Gli3	R&D Systems (Minnneapolis, MN, USA)	AF3690	WB (1:1000)
Anti-HA	BioLegend (San Diego, CA, USA)		IF, WB (1:1000)
Anti-PARP	Cell Signaling Technologies (Danvers, MA, USA)	9532	WB (1:1000)
Anti-GAPDH	Thermo Fisher (Waltham, MA, USA)	PA1-988	WB (1:1000)
Anti-α-tubulin	Sigma (Darmstadt, Germany)	T6199	WB (1:1000)
Anti-acetylated tubulin	Sigma (Darmstadt, Germany)	T6793	WB (1:1000)
Anti-Lamin A/C	Thermo Fisher (Waltham, MA, USA)	MA5–35284	WB (1:1000)
Anti-Arl13b	Proteintech (San Diego, CA, USA)	17711-1-AP	IF (1:1000)
Secondary antibodies			
Anti-mouse HRP	BioLegend (San Diego, CA, USA)	405306	WB (1:2500)
Anti-goat HRP	Sigma (Darmstadt, Germany)	A5420	WB (1:2500)
Anti-rabbit HRP	BioLegend (San Diego, CA, USA)	406401	WB (1:2500)
Anti-mouse Alexa-488	Jackson Immunoresearch (West Grove, PA, USA)	715–545-151	IF (1:1000)
Anti-rabbit Alexa-594	Jackson Immunoresearch (West Grove, PA, USA)	711–585-152	IF (1:1000)

**Table 2:** Oligonucleotides used for CRISPR/Cas9 mutagenesis and qPCR primers.

Name	Sequence
sgRNA-Non-target	GCGAGGTATTCGGCTCCGCG
sgRNA-Sufu	ATACCAGTACTTGACGATAG
sgRNA2-Gli1	GGCTGGACTCCATAGGG
sgRNA1-Gli2	GCTTGCGGCTCAGTCGTG
sgRNA1-Gli3	CCTCGACGTCTAGTGATGAG
sgRNA2-Btbd9	CAGCACATACAAGTTAACTT
sgRNA2- Btbd10	TGGGCGCTTGCTACCATGAG
sgRNA1-Cul1	ATTCCATTCAGCACTTTGC
sgRNA2-Cul1	TGCCTACCTCAATAGACAT
sgRNA4-Cul1	CATCAGTCCAACCAAGCCC
sgRNA4-Cul3	TAGAAAATGTCTACAATTT
sgRNA5-Cul3	ACCTAAAATCATTAACATC
sgRNA1-Kctd3	AGTGAGGTAAACGCTCAGCG
sgRNA1-Klhl9	CTTTCTACAGATCTTGC
sgRNA2-Klhl9	TCTGCCAGTGATTATTTCA
sgRNA1-Rbx1	GTTATCAACCACAATGTCCC
sgRNA2-Rbx1	GGCCTGACATTCGATACCTG
sgRNA3-Rbx1	CTCCTTGCAGTGGAATGCAG
sgRNA4-Rbx1	GCGGCGGCGATGGATG
sgRNA5-Rbx1	CCGCTGTTGGTGCCGCTGG
sgRNA1-Stub1	CCGCCTCCGGGTACTTG
sgRNA2-Stub1	CAATGCGAAGGGCACT
sgRNA1-Ube2l3	TCATTCACCAGTGCTATGA
sgRNA2-Ube2l3	ACAACCCTCCATATGATA
sgRNA1 Myh1	GCTGCCAGTGTATAACGCAG
sgRNA1 Myh8	CCAGATTATAACAAGAACCC
sgRNA2 Myh8	GAATCCACTTCGGTACCACG
sgRNA2 Mov10	CAAGACTGTCACATTAGTGG
sgRNA1 Peak1	AGTGCAACCTACAGCAACCT
sgRNA2 Peak1	CTGATGCAAAACCTAAACGC
sgRNA1 Scyl2	TCTGCAT CGGGTCTCACGGT
sgRNA1 Med12	CAAACAGTACGACCAACCGC
sgRNA2 Med12	CTGTTGGAAAACCTCGATTG
sgRNA1 Nipbl	CTCCCATCTCCTTTACCTGC
qPCR GAPDH F (Ms)	AGCCTCGTCCCGTAGACAAAAT
qPCR GAPDH R (Ms)	CCGTGAGTGGAGTCATACTGGA
qPCR Gli1 F (Ms)	CCAAGCCAACTTTATGTCAGGG
qPCR Gli1 R (Ms)	AGCCCGCTTCTTTGTTAATTTGA
qPCR Gli1 3′UTR F (Ms)	GCCTCTCCCACATACTAGAAATC
qPCR Gli1 3′UTR R (Ms)	CATTGGATTGAACATGGCGTC
qPCR Gli2 F (Ms)	CCCACTCCAGCCAAGTTG
qPCR Gli2 R (Ms)	GCAGAAGTCTCCATCTCAGAG
qPCR Hes1 F (Ms)	GGCGAAGGGCAAGAATAAATG
qPCR Hes1 R (Ms)	GTGCTTCACAGTCATTTCCAG
qPCR Ptch1 F (Ms)	CTGCCTGTCCTCTTATCCTTC
qPCR Ptch1 R (Ms)	AGACCCATTGTTCGTGTGAC
qPCR GAPDH F (Hs)	ACATCGCTCAGACACCAT
qPCR GAPDH R (Hs)	TGTAGTTGAGGTCAATGAAGGG
qPCR Gli1 F (Hs)	TCTGGACATACCCCACCTCCCTCTG
qPCR Gli1 R (Hs)	ACTGCAGCTCCCCCAATTTTTCTGG

## Data Availability

All data supporting the findings of this study are available within the paper and its Supplementary Data. Raw spectrometry data will be available on request.

## Supplementary Materials

The following supporting information can be downloaded at: https://www.mdpi.com/article/10.3390/cells13171496/s1; Figure S1. Proteasomal inhibition downregulates the Hedgehog pathway. (A) Protein levels of Gli1, Gli2, and poly-ubiquitinated proteins were analyzed in NIH-3T3 cells expressing constitutively active Gli2 mutant Gli2(P1-6A) treated with proteasome inhibitors as indicated (Bort.—bortezomib). (B) Endogenous protein levels of Gli1, Gli2, Gli3FL, Gli3R, and poly-ubiquitinated proteins were analyzed in NIH-3T3 cells treated with proteasome inhibitors as indicated (Epox.—epoxomicin 30 nM, MG-132 1 or 3 µM; 14 or 24 h). Tubulin and β-actin were used as loading control. Relative protein level normalized to tubulin and compared to non-treated sample is shown below respective bands. (C) Densitometric quantification of Gli1 protein level from panel (B). Error bars represent SD from four replicates. *p*-value: *** *p* < 0.001. (D) PARP cleavage was analyzed to assess the cytotoxicity of proteasome inhibitors treatment in NIH-3T3 cells. (E) Trypan blue exclusion assay was used to evaluate cell viability upon proteasome inhibitors treatment. Bars represent the percentage of trypan blue-positive cells. Error bars represent SD from two replicates. *p*-value: ** *p* < 0.01. (F) Luciferase reporter assay was performed in serum-starved NIH-3T3 cells expressing constitutively active Gli2 mutant Gli2(P1-6A) after 24 h treatment with proteasome inhibitor carfilzomib (Carf) as indicated. Error bars represent SD from three replicates. *p*-value: ** *p* < 0.01. (G) Hh luciferase reporter assay was performed in serum-starved NIH-3T3 cells after treatment with proteasome inhibitor lactacystin as indicated. Pathway activity was stimulated as in Figure 1A. Error bars represent SD from five replicates. *p*-value: *** *p* < 0.001. (H) mRNA level of Hh target gene *Gli1* was analyzed in serum-starved human medulloblastoma Daoy cell line after 24 h treatment with proteasome inhibitor epoxomicin (Epox) and SAG, as indicated. Error bars represent SD from three replicates. *p*-value: *** *p* < 0.001, * *p* < 0.05. Figure S2. The specificity of the Hedgehog pathway downregulation upon proteasomal inhibition. (A) Protein levels of Gli1, acetylated tubulin, and Gli2(P1-6A) mutant were analyzed after 24 h treatment with proteasome inhibitors or Taxol in serum-starved NIH-3T3 cells as indicated (Bort.—Bortezomib). GAPDH was used as a loading control. (B) mRNA level of Hh target genes *Gli1* and *Ptch1* were analyzed in serum-starved NIH-3T3 expressing constitutively active Gli2(P1-6A) mutant after 24 h treatment with a proteasome inhibitor (Bort.—bortezomib) or Taxol. Error bars represent SD from four replicates. *p*-value: *** *p* < 0.001, ** *p* < 0.01. (C) Protein level of HA-tagged Gli2(P1-6A) mutant was analyzed after 6 h treatment with epoxomicin in serum-starved NIH-3T3 within total lysate (Total) and nuclear (Nuc) and cytosolic (Cyto) fractions (densitometric quantification below the bands). Lamin A and tubulin were used as loading controls for nuclear and cytosolic fractions respectively. (D) Protein level of HA-tagged Gli2(P1-6A) mutant was analyzed after 6 h treatment with indicated proteasome inhibitors (X—non-treated, B30—bortezomib 30 nM, M3—MG-132 µM) in serum-starved NIH-3T3 cells within nuclear (N, Nuc) and cytosolic (C, Cyto) fractions (densitometric quantification below the bands). Parp and tubulin were used as loading controls for nuclear and cytosolic fractions respectively. (E) Protein levels of full-length (FL) Gli3 and Gli3R were analyzed in the WT NIH-3T3 cell line upon Gli3 KO in the clonal cell line. Tubulin was used as a loading control. (F) Protein levels of Gli1 and Gli3 repressor (Gli3R) were analyzed in serum-starved NIH-3T3 expressing constitutively active Gli2(P1-6A) mutant as in Figure 2A (Epox—epoxomicin) to assess Gli3R stabilization. Tubulin was used as a loading control. (G) Protein level of Gli2 was analyzed in the WT NIH-3T3 cell line upon Gli2 KO in a clonal cell line. Tubulin was used as a loading control. (H) Protein level of Gli1 was analyzed in WT NIH-3T3 and cilia-less DN Kif3a cell lines upon Gli1 KO. Lamin A was used as a loading control. (I) Protein level of Gli3 was analyzed in the NIH-3T3 Gli2 KO cells upon Gli3 KO in a clonal cell line. Tubulin was used as a loading control. (J) mRNA level of Hh target gene *Gli1* was analyzed in serum-starved WT NIH-3T3 or Gli2/3 KO clonal cell line upon the rescue with the Gli2(P1-6A) constitutively active. Pathway activity was stimulated in WT NIH-3T3 as in Figure 1A. Error bars represent SD from four replicates. *p*-value: **** *p* < 0.0001, * *p* < 0.05. (K) Protein levels of Gli1, Gli2, and Gli3 were analyzed in Gli1 KO, Gli1/2 double KO, and Gli1/3 double KO cells. WT NIH-3T3 cell line was transduced with indicated sgRNAs (NT—non-targeting control). Tubulin was used a as loading control. Gli1, Gli2, and Gli3 protein levels were normalized to tubulin and the level relative to NT control is shown below the respective bands. Figure S3. (A) STRING network of high-confidence Gli2(P1-6A) interactors from pooled data sets of control and epoxomicin-treated cells. The intensity of the blue color represents the relative total abundance in the control and epoxomicin-treated cells. Pie charts represent the ratio between control and epoxomicin-treated cells. (B) mRNA level of Hh target gene *Gli1* was analyzed in serum-starved NIH-3T3 expressing constitutively active Gli2(P1-6A) mutant upon knockout of indicated genes after 24 h treatment with 20 nM epoxomicin. Error bars represent SD from three replicates. All samples were compared against non-treated control. *p*-value: *** *p* < 0.001, ** *p* < 0.01. Figure S4. E3 ubiquitin ligase screening. Immunofluorescent staining of starved NIH-3T3 GFP reporter cell line upon KO of indicated genes was done to assess the effect of gene KO on ciliogenesis. Arl13b was used as a marker of primary cilium. Scale bar, 5 µm. Figure S5. Efficiency of CRISPR KO for selected genes. (A) Protein level of V5-tagged Rbx1 was analyzed using Western blot in Hek293 cells expressing Rbx1-V5 transfected with non-targeting control or indicated sgRNA against Rbx1. Samples on the left and the right membranes represent technical replicates. Tubulin was used as a loading control. (B) DNA from Cul3 sg4 KO cells was analyzed using TIDE software (version 3.3.0). (C) DNA from Kctd3 sg1 KO cells was analyzed using TIDE software. (D) DNA from Btbd9 sg2 KO cells was analyzed using TIDE software. Table S1. Proteins enriched in the epoxomicin-treated cells form mass spectrometry analysis. Rows are ordered from the most enriched proteins. Proteins were identified as enriched if their Epox over DMSO ratio was greater or equal to 1.5. Table S2. Proteins depleted in the epoxomicin-treated cells form mass spectrometry analysis. Rows are ordered from the most depleted proteins. Proteins were identified as depleted if their Epox over DMSO ratio was smaller or equal to 0.75. Table S3. Panther analysis of subsets of enriched and depleted proteins in epoxomicin-treated cells.
