# Supplementary material for: The Proteasome and Cul3-Dependent Protein Ubiquitination Is Required for Gli Protein-Mediated Activation of Gene Expression in the Hedgehog Pathway
Source: Cells. 2024 Sep 6;13(17):1496. doi: 10.3390/cells13171496 (PMC11394618; doi:10.3390/cells13171496)
Supplement: Supplementary file 1 [file cells-13-01496-s001.zip › cells-3014183-supplementary.pdf]

cells

Figure S1

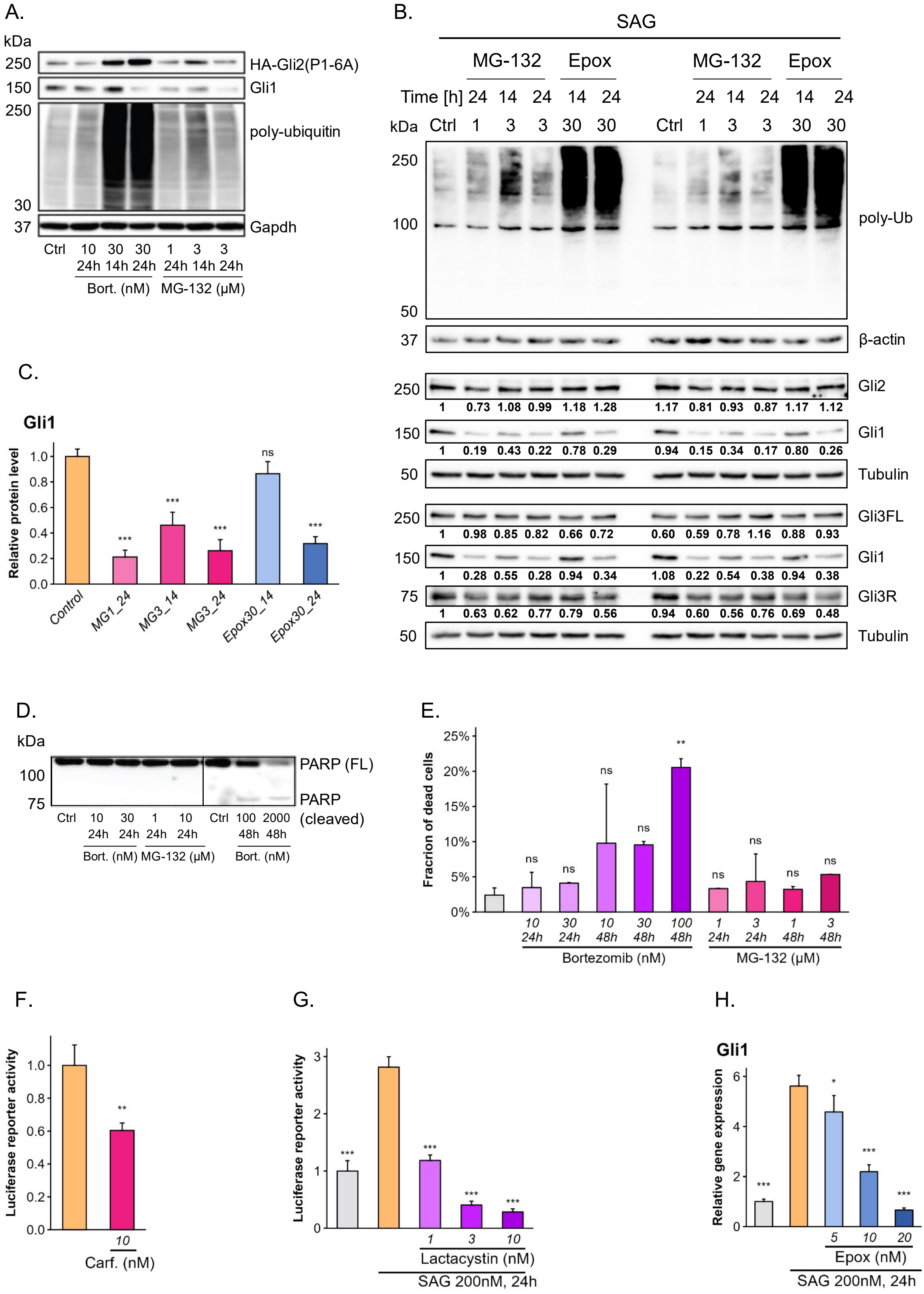

Figure S1. Proteasomal inhibition downregulates the Hedgehog pathway. (A) Protein levels of Gli1, Gli2, and poly-ubiquitinated proteins were analyzed in NIH-3T3 cells expressing constitutively active Gli2 mutant Gli2(P1-6A) treated with proteasome inhibitors as indicated (Bort.—bortezomib). (B) Endogenous protein levels of Gli1, Gli2, Gli3FL, Gli3R, and poly-ubiquitinated proteins were analyzed in NIH-3T3 cells treated with proteasome inhibitors as indicated (Epox.—epoxomicin 30 nM, MG-132 1 or 3 μM; 14 or 24 h). Tubulin and β-actin were used as loading control. Relative protein level normalized to tubulin and compared to non-treated sample is shown below respective bands. (C) Densitometric quantification of Gli1 protein level from panel (B). Error bars represent SD from four replicates. *p*-value: *** $p < 0.001$. (D) PARP cleavage was analyzed to assess the cytotoxicity of proteasome inhibitors treatment in NIH-3T3 cells. (E) Trypan blue exclusion assay was used to evaluate cell viability upon proteasome inhibitors treatment. Bars represent the percentage of trypan blue-positive cells. Error bars represent SD from two replicates. *p*-value: ** $p < 0.01$. (F) Luciferase reporter assay was performed in serum-starved NIH-3T3 cells expressing constitutively active Gli2 mutant Gli2(P1-6A) after 24 h treatment with proteasome inhibitor carfilzomib (Carf) as indicated. Error bars represent SD from three replicates. *p*-value: ** $p < 0.01$. (G) Hh luciferase reporter assay was performed in serum-starved NIH-3T3 cells after treatment with proteasome inhibitor lactacystin as indicated. Pathway activity was stimulated as in Figure 1A. Error bars represent SD from five replicates. *p*-value: *** $p < 0.001$. (H) mRNA level of Hh target gene *Gli1* was analyzed in serum-starved human medulloblastoma Daoy cell line after 24 h treatment with proteasome inhibitor epoxomicin (Epox) and SAG, as indicated. Error bars represent SD from three replicates. *p*-value: *** $p < 0.001$, * $p < 0.05$

Figure S2

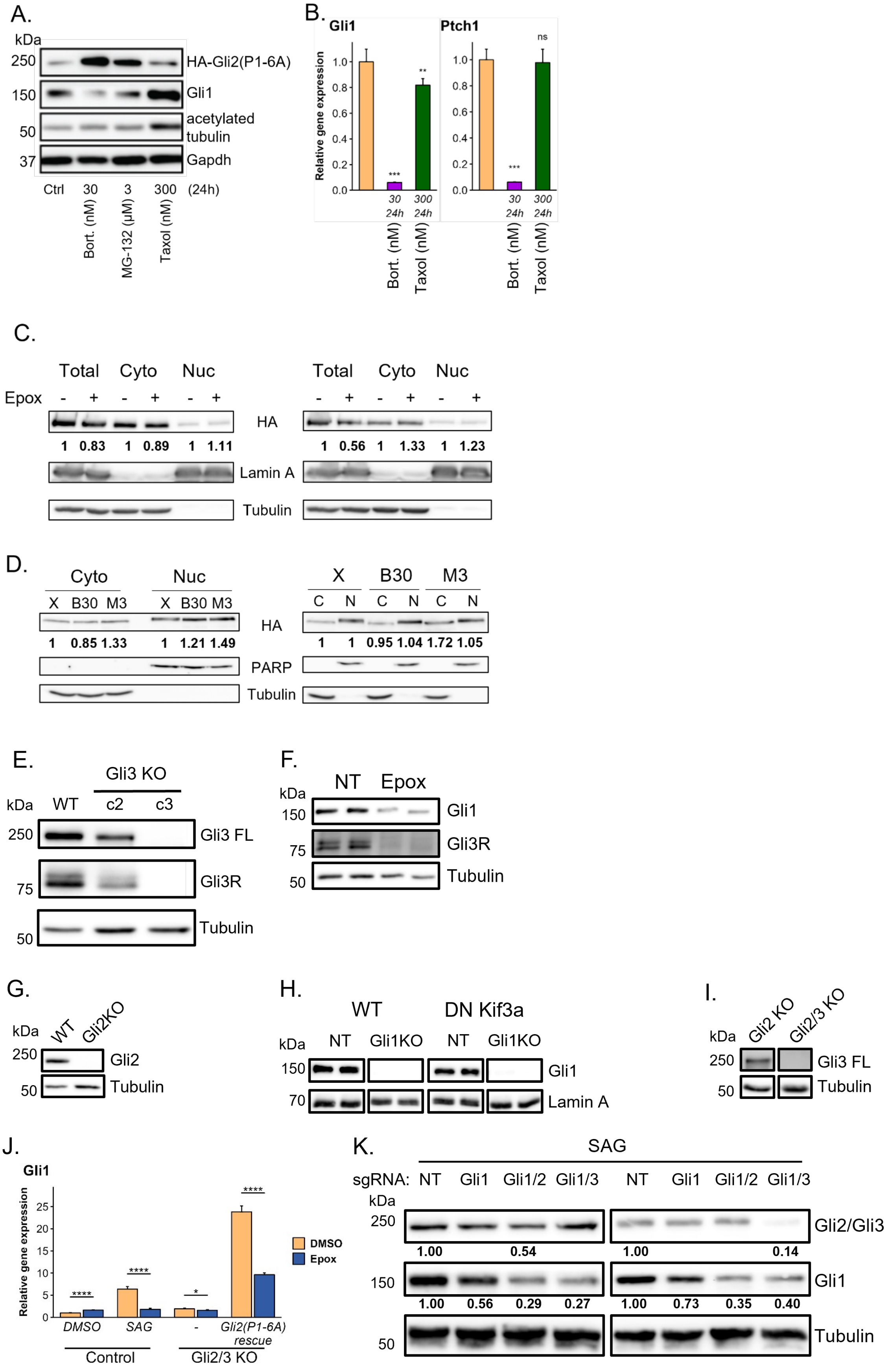
A.
kDa
250
150
50
37
HA-Gli2(P1-6A)
Gli1
acetylated tubulin
Gapdh
Ctrl 30 3 300 (24h)
Bort. (nM)
MG-132 (µM)
Taxol (nM)
B.
Gli1
Ptch1
Relative gene expression
Bort. (nM)
Taxol (nM)
C.
Total Cyto Nuc
Epox
HA
1 0.83 1 0.89 1 1.11
1 0.56 1 1.33 1 1.23
Lamin A
Tubulin
D.
Cyto Nuc
X B30 M3
HA
1 0.85 1.33 1 1.21 1.49
1 1 0.95 1.04 1.72 1.05
PARP
Tubulin
E.
Gli3 KO
WT c2 c3
Gli3 FL
Gli3R
Tubulin
F.
NT Epox
Gli1
Gli3R
Tubulin
G.
WT Gli2KO
Gli2
Tubulin
H.
WT DN Kif3a
NT Gli1KO
Gli1
Lamin A
I.
Gli2 KO Gli2/3 KO
Gli3 FL
Tubulin
J.
Gli1
Relative gene expression
DMSO
Epox
DMSO SAG - Gli2(P1-6A) rescue
Control Gli2/3 KO
K.
SAG
sgRNA: NT Gli1 Gli1/2 Gli1/3 NT Gli1 Gli1/2 Gli1/3
Gli2/Gli3
1.00 0.54 1.00 0.14
Gli1
1.00 0.56 0.29 0.27 1.00 0.73 0.35 0.40
Tubulin

Figure S2. The specificity of the Hedgehog pathway downregulation upon proteasomal inhibition. (A) Protein levels of Gli1, acetylated tubulin, and Gli2(P1-6A) mutant were analyzed after 24 h treatment with proteasome inhibitors or Taxol in serum-starved NIH-3T3 cells as indicated (Bort.—Bortezomib). GAPDH was used as a loading control. (B) mRNA level of Hh target genes *Gli1* and *Ptch1* were analyzed in serum-starved NIH-3T3 expressing constitutively active Gli2(P1-6A) mutant after 24 h treatment with a proteasome inhibitor (Bort.—bortezomib) or Taxol. Error bars represent SD from four replicates. *p*-value: *** $p < 0.001$, ** $p < 0.01$. (C) Protein level of HA-tagged Gli2(P1-6A) mutant was analyzed after 6 h treatment with epoxomicin in serum-starved NIH-3T3 within total lysate (Total) and nuclear (Nuc) and cytosolic (Cyto) fractions (densitometric quantification below the bands). Lamin A and tubulin were used as loading controls for nuclear and cytosolic fractions respectively. (D) Protein level of HA-tagged Gli2(P1-6A) mutant was analyzed after 6 h treatment with indicated proteasome inhibitors (X—non-treated, B30—bortezomib 30 nM, M3—MG-132 μM) in serum-starved NIH-3T3 cells within nuclear (N, Nuc) and cytosolic (C, Cyto) fractions (densitometric quantification below the bands). Parp and tubulin were used as loading controls for nuclear and cytosolic fractions respectively. (E) Protein levels of full-length (FL) Gli3 and Gli3R were analyzed in the WT NIH-3T3 cell line upon Gli3 KO in the clonal cell line. Tubulin was used as a loading control. (F) Protein levels of Gli1 and Gli3 repressor (Gli3R) were analyzed in serum-starved NIH-3T3 expressing constitutively active Gli2(P1-6A) mutant as in Figure 2A (Epox—epoxomicin) to assess Gli3R stabilization. Tubulin was used as a loading control. (G) Protein level of Gli2 was analyzed in the WT NIH-3T3 cell line upon Gli2 KO in a clonal cell line. Tubulin was used as a loading control. (H) Protein level of Gli1 was analyzed in WT NIH-3T3 and cilia-less DN Kif3a cell lines upon Gli1 KO. Lamin A was used as a loading control. (I) Protein level of Gli3 was analyzed in the NIH-3T3 Gli2 KO cells upon Gli3 KO in a clonal cell line. Tubulin was used as a loading control. (J) mRNA level of Hh target gene *Gli1* was analyzed in serum-starved WT NIH-3T3 or Gli2/3 KO clonal cell line upon the rescue with the Gli2(P1-6A) constitutively active. Pathway activity was stimulated in WT NIH-3T3 as in Figure 1A. Error bars represent SD from four replicates. *p*-value: **** $p < 0.0001$, * $p < 0.05$. (K) Protein levels of Gli1, Gli2, and Gli3 were analyzed in Gli1 KO, Gli1/2 double KO, and Gli1/3 double KO cells. WT NIH-3T3 cell line was transduced with indicated sgRNAs (NT—non-targeting control). Tubulin was used a as loading control. Gli1, Gli2, and Gli3 protein levels were normalized to tubulin and the level relative to NT control is shown below the respective bands.

Figure S3

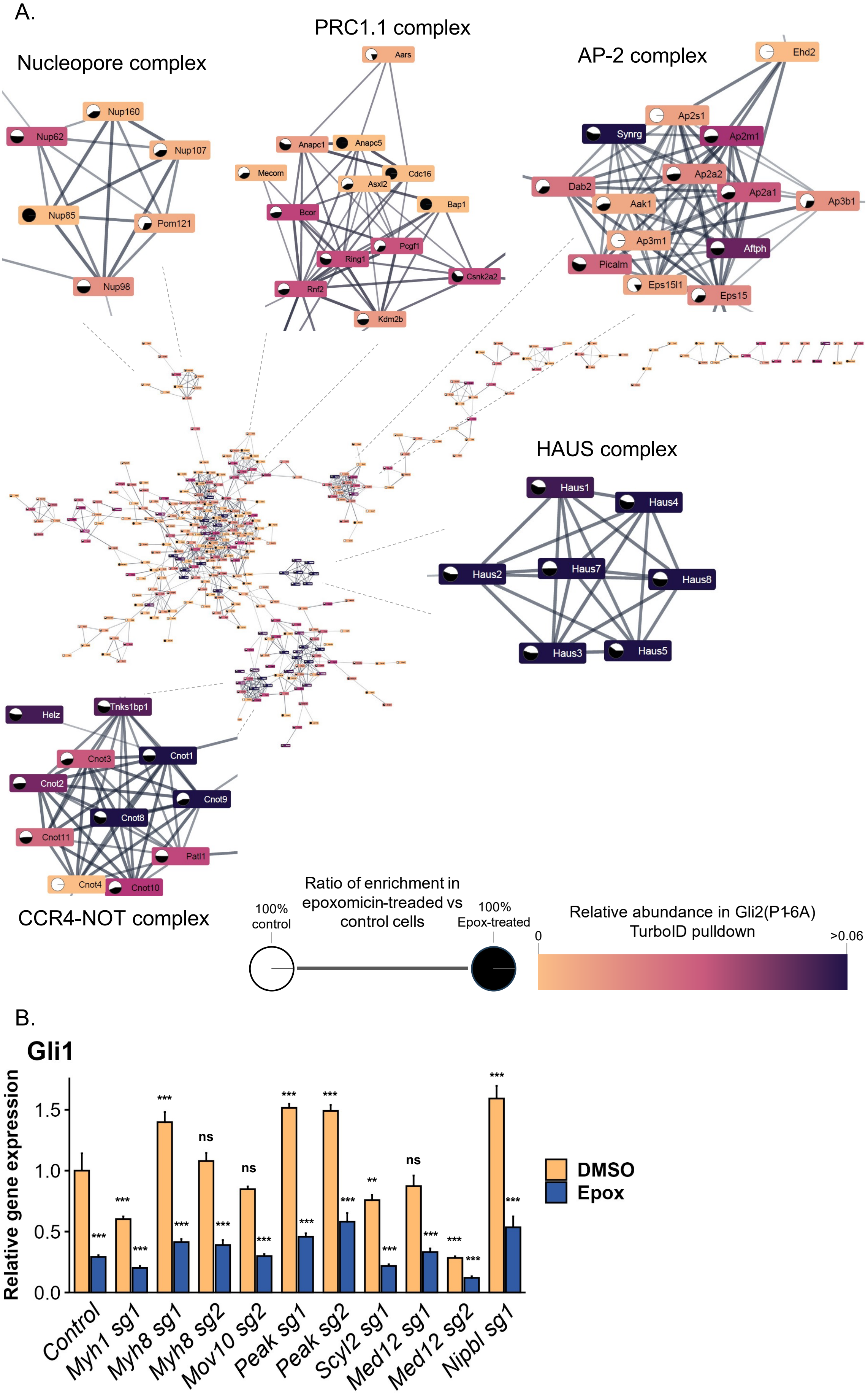
A.
PRC1.1 complex
Nucleopore complex
AP-2 complex
Aars
Ehd2
Nup160
Nup62
Nup107
Nup85
Pom121
Nup98
Anapc1
Anapc5
Mecom
Cdc16
Asxl2
Bap1
Bcor
Pcgf1
Ring1
Csnk2a2
Rnf2
Kdm2b
Ap2s1
Synrg
Ap2m1
Ap2a2
Dab2
Ap2a1
Aak1
Ap3b1
Ap3m1
Aftph
Picalm
Eps15l1
Eps15
HAUS complex
Haus1
Haus4
Haus2
Haus7
Haus8
Haus3
Haus5
Helz
Tnks1bp1
Cnot3
Cnot1
Cnot2
Cnot9
Cnot8
Cnot11
Patl1
Cnot4
Cnot10
CCR4-NOT complex
Ratio of enrichment in epoxomicin-treaded vs control cells
100% control
100% Epox-treated
Relative abundance in Gli2(P1-6A) TurboID pulldown
0
>0.06
B.
Gli1
Relative gene expression
0.0
0.5
1.0
1.5
DMSO
Epox
Control
Myh1 sg1
Myh8 sg1
Myh8 sg2
Mov10 sg2
Peak sg1
Peak sg2
Scyl2 sg1
Med12 sg1
Med12 sg2
Nipbl sg1

Figure S3. (A) STRING network of high-confidence Gli2(P1-6A) interactors from pooled data sets of control and epoxomicin-treated cells. The intensity of the blue color represents the relative total abundance in the control and epoxomicin-treated cells. Pie charts represent the ratio between control and epoxomicin-treated cells. (B) mRNA level of Hh target gene *Gli1* was analyzed in serum-starved NIH-3T3 expressing constitutively active Gli2(P1-6A) mutant upon knockout of indicated genes after 24 h treatment with 20 nM epoxomicin. Error bars represent SD from three replicates. All samples were compared against non-treated control. *p*-value: *** $p < 0.001$, ** $p < 0.01$.

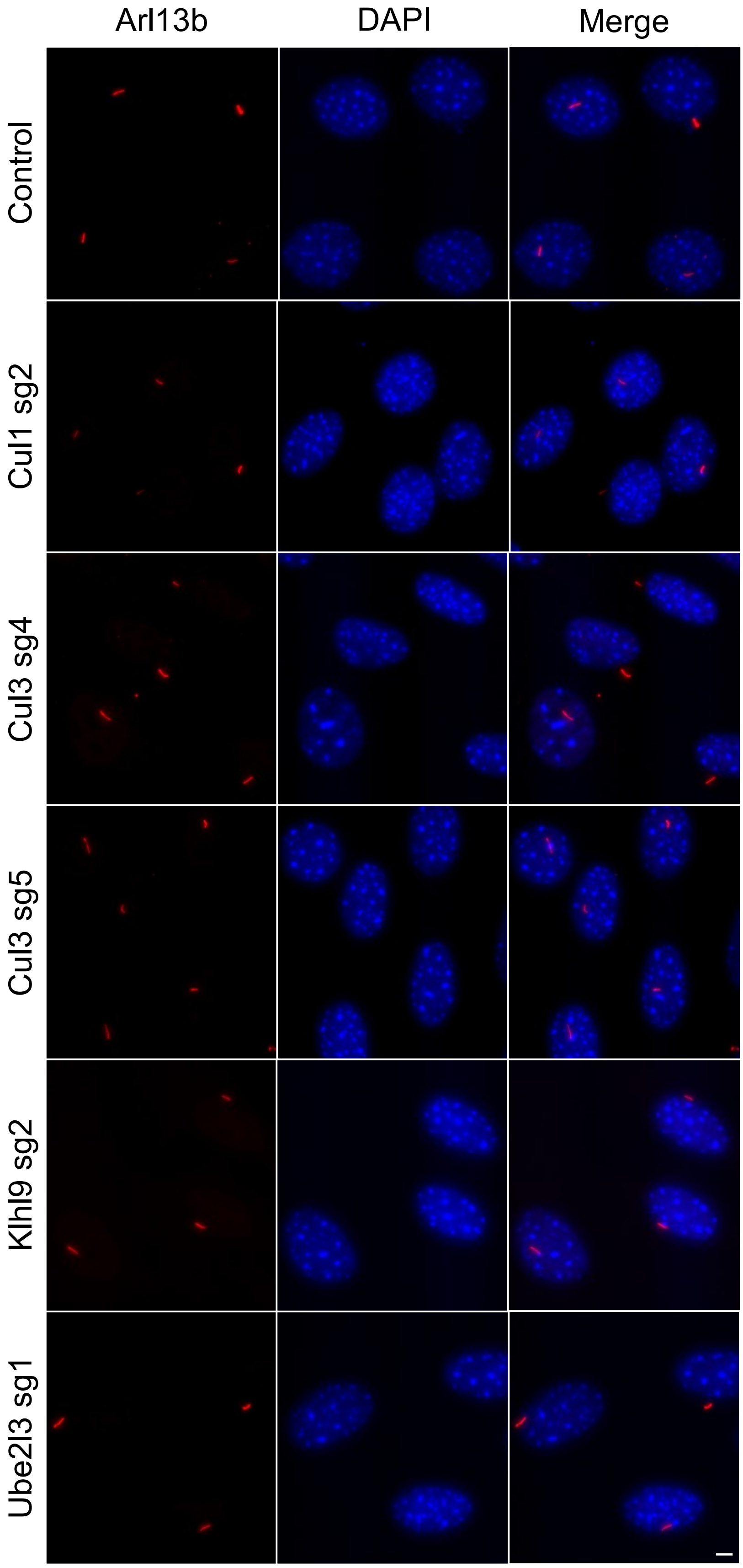

Figure S4. E3 ubiquitin ligase screening. Immunofluorescent staining of starved NIH-3T3 GFP reporter cell line upon KO of indicated genes was done to assess the effect of gene KO on ciliogenesis. Arl13b was used as a marker of primary cilium. Scale bar, 5 μm.

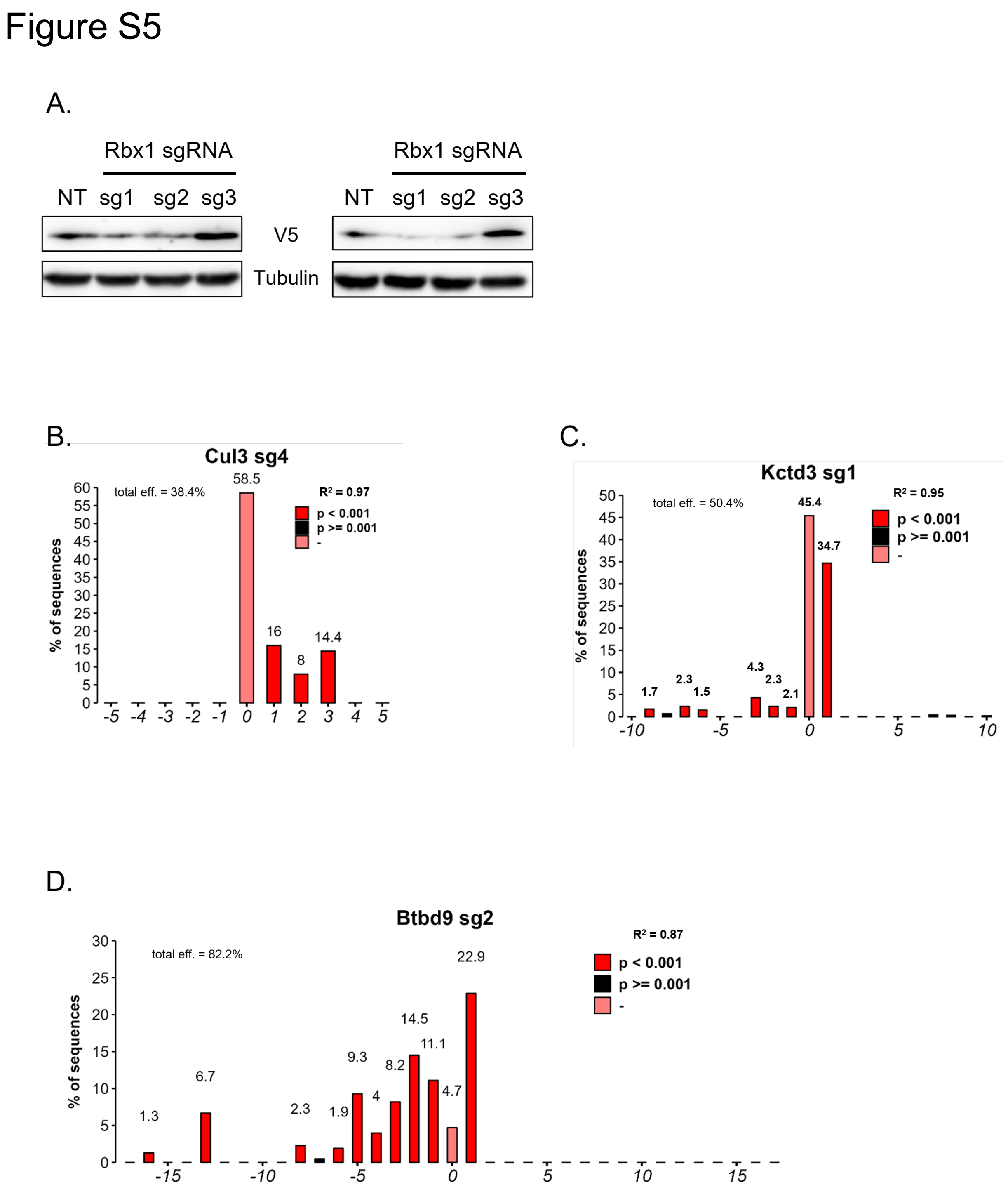

Figure S5. Efficiency of CRISPR KO for selected genes. (A) Protein level of V5-tagged Rbx1 was analyzed using Western blot in Hek293 cells expressing Rbx1-V5 transfected with non-targeting control or indicated sgRNA against Rbx1. Samples on the left and the right membranes represent technical replicates. Tubulin was used as a loading control. (B) DNA from Cul3 sg4 KO cells was analyzed using TIDE software (version 3.3.0). (C) DNA from Kctd3 sg1 KO cells was analyzed using TIDE software. (D) DNA from Btbd9 sg2 KO cells was analyzed using TIDE software.

Table S1. Proteins enriched in the epoxomicin-treated cells form mass spectrometry analysis. Rows are ordered from the most enriched proteins. Proteins were identified as enriched if their Epox over DMSO ratio was greater or equal to 1.5.

ID	Full protein name	Gene	Mean Gli2(P1-6A) DMSO normalized	Mean Gli2(P1-6A) Epox normalized	Gli2(P1-6A) DMSO peptide abundance relative to control	Gli2(P1-6A) Epox peptide abundance relative to control	Relative enrichment in epoxomicin-treated cells
P68134	Actin, alpha skeletal muscle	Acta1	0.00	15.48	0.00	154.79	154.79
Q5SX39	Myosin-4	Myh4	0.00	11.34	0.00	113.45	113.45
Q5SX40	Myosin-1	Myh1	0.00	8.40	0.00	84.03	84.03
P13542	Myosin-8	Myh8	0.00	6.30	0.00	63.03	63.03
Q8CFE4	SCY1-like protein 2	Scyl2	3.78	10.44	37.84	104.37	2.76
P07901	Heat shock protein HSP 90-alpha	Hsp90aa1	3.12	7.43	0.78	1.86	2.38
Q69ZN7	Myoferlin	Myof	2.34	5.24	23.38	52.35	2.24
Q8BMD7	MORC family CW-type zinc finger protein 4	Morc4	3.23	7.11	32.30	71.09	2.20
Q925H1	Zinc finger transcription factor Trps1	Trps1	2.84	6.17	28.38	61.72	2.17
Q69Z38	Inactive tyrosine-protein kinase PEAK1	Peak1	3.28	7.11	32.84	71.09	2.16
Q6ZQ58	La-related protein 1	Larp1	4.62	9.63	46.22	96.30	2.08
O35382	Exocyst complex component 4	Exoc4	4.23	8.47	42.30	84.66	2.00
Q6NZQ4	PAX-interacting protein 1	Paxip1	2.68	5.33	26.76	53.32	1.99
Q60520	Paired amphipathic helix protein Sin3a	Sin3a	4.68	8.89	46.76	88.86	1.90
Q05D44	Eukaryotic translation initiation factor 5B	Eif5b	2.84	5.33	0.77	1.45	1.88
A2AGH6	Mediator of RNA polymerase II transcription subunit 12	Med12	2.84	5.24	28.38	52.35	1.84
P23249	Putative helicase MOV-10	Mov10	6.01	10.66	60.15	106.64	1.77
Q8BGC4	Prostaglandin reductase-3	Ptgr3	3.28	5.75	32.84	57.52	1.75
P18760	Cofilin-1	Cfl1	6.18	10.66	0.95	1.64	1.73
A2A870	Fas-binding factor 1	Fbf1	4.84	8.05	48.38	80.46	1.66
P46471	26S proteasome regulatory subunit 7	Psmc2	3.78	6.17	1.19	1.94	1.63
Q6KCD5	Nipped-B-like protein	Nipbl	4.68	7.11	46.76	71.09	1.52

Table S2. Proteins depleted in the epoxomicin-treated cells form mass spectrometry analysis. Rows are ordered from the most depleted proteins. Proteins were identified as depleted if their Epox over DMSO ratio was smaller or equal to 0.75.

ID	Full protein name	Gene	Mean Gli2(P1-6A) DMSO normalized	Mean Gli2(P1-6A) Epox normalized	Gli2(P1-6A) DMSO peptide abundance relative to control	Gli2(P1-6A) Epox peptide abundance relative to control	Relative enrichment in epoxomicin-treated cells
P62984	Ubiquitin-ribosomal protein eL40 fusion protein	Uba52	6.12	0.00	1.44	0.00	0.00
Q6P5D8	Structural maintenance of chromosomes flexible hinge domain-containing protein 1	Smchd1	5.18	0.00	51.76	0.00	0.00
Q8VEM8	Solute carrier family 25 member 3	Slc25a3	7.12	0.84	1.97	0.23	0.12
Q6PDI5	Proteasome adapter and scaffold protein ECM29	Ecpas	5.62	0.84	56.22	8.40	0.15
Q91WQ3	Tyrosine--tRNA ligase, cytoplasmic	Yars1	8.85	1.68	1.39	0.26	0.19
Q6PB66	Leucine-rich PPR motif-containing protein, mitochondrial	Lrpprc	9.46	2.10	8.42	1.87	0.22
Q99LE6	ATP-binding cassette sub-family F member 2	Abcf2	7.01	1.68	4.16	1.00	0.24
P46978	Dolichyl-diphosphooligosaccharide--protein glycosyltransferase subunit STT3A	Stt3a	5.12	1.26	4.56	1.12	0.25
P47806	Zinc finger protein GLI1	Gli1	7.07	2.10	70.69	21.01	0.30
Q3UPL0	Protein transport protein Sec31A	Sec31a	6.62	2.10	3.12	0.99	0.32
B2RX14	Terminal uridylyltransferase 4	Tut4	8.96	2.94	89.61	29.41	0.33
Q61753	D-3-phosphoglycerate dehydrogenase	Phgdh	7.62	2.52	1.44	0.47	0.33
Q6ZQ38	Cullin-associated NEDD8-dissociated protein 1	Cand1	6.18	2.10	4.12	1.40	0.34
Q60838	Segment polarity protein dishevelled homolog DVL-2	Dvl2	6.07	2.10	60.69	21.01	0.35
Q8CDG3	Deubiquitinating protein VCPIP1	Vcpip1	9.01	3.36	90.15	33.61	0.37
P54071	Isocitrate dehydrogenase [NADP], mitochondrial	Idh2	6.62	2.52	3.31	1.26	0.38
Q9QXZ0	Microtubule-actin cross-linking factor 1, isoforms 1/2/3/4	Macf1	9.46	3.78	94.61	37.82	0.40
Q9EQH3	Vacuolar protein sorting-associated protein 35	Vps35	5.18	2.10	51.76	21.01	0.41
P39053	Dynamin-1	Dnm1	5.18	2.10	1.66	0.67	0.41
Q8C0C7	Phenylalanine--tRNA ligase alpha subunit	Farsa	5.18	2.10	4.61	1.87	0.41
Q9Z1T1	AP-3 complex subunit beta-1	Ap3b1	7.12	2.94	6.34	2.62	0.41
P10630	Eukaryotic initiation factor 4A-II	Eif4a2	11.74	5.04	117.45	50.42	0.43
P80317	T-complex protein 1 subunit zeta	Cct6a	9.41	4.39	2.51	1.17	0.47
A2A690	Protein TANC2	Tanc2	5.34	2.52	53.38	25.21	0.47
O08553	Dihydropyrimidinase-related protein 2	Dpysl2	6.18	2.94	61.76	29.41	0.48
Q9JJG0	Transforming acidic coiled-coil-containing protein 2	Tacc2	5.23	2.52	52.30	25.21	0.48
Q99JR8	SWI/SNF-related matrix-associated actin-dependent regulator of chromatin subfamily D member 2	Smarcd2	5.62	2.81	56.22	28.11	0.50
P17225	Polypyrimidine tract-binding protein 1	Ptbp1	5.62	2.94	1.80	0.94	0.52

P47911	Large ribosomal subunit protein eL6	Rpl6	5.62	2.94	1.32	0.69	0.52
P42567	Epidermal growth factor receptor substrate 15	Eps15	6.23	3.36	62.30	33.61	0.54
Q91VC3	Eukaryotic initiation factor 4A-III	Eif4a3	8.07	4.39	2.58	1.41	0.54
Q9CR62	Mitochondrial 2-oxoglutarate/malate carrier protein	Slc25a11	6.12	3.36	1.66	0.91	0.55
P98078	Disabled homolog 2	Dab2	7.07	3.97	70.69	39.75	0.56
Q99K48	Non-POU domain-containing octamer-binding protein	Nono	9.80	5.66	1.85	1.07	0.58
O08749	Dihydrolipoyl dehydrogenase, mitochondrial	Dld	7.57	4.39	1.57	0.91	0.58
Q8BRH4	Histone-lysine N-methyltransferase 2C	Kmt2c	6.46	3.78	64.61	37.82	0.59
O09000	Nuclear receptor coactivator 3	Ncoa3	9.24	5.75	92.45	57.52	0.62
Q8K1M6	Dynamin-1-like protein	Dnm1l	8.57	5.33	8.57	5.33	0.62
Q6A065	Centrosomal protein of 170 kDa	Cep170	8.51	5.33	85.15	53.32	0.63
Q61024	Asparagine synthetase [glutamine-hydrolyzing]	Asns	5.23	3.36	4.65	2.99	0.64
Q9EPK7	Exportin-7	Xpo7	6.07	3.97	60.69	39.75	0.65
E9Q7G0	Nuclear mitotic apparatus protein 1	Numa1	13.24	8.69	1.80	1.18	0.66
O35643	AP-1 complex subunit beta-1	Ap1b1	8.01	5.33	3.77	2.51	0.67
Q8BJ34	Meiosis regulator and mRNA stability factor 1	Marf1	8.01	5.33	80.15	53.32	0.67
Q9JIF7	Coatomer subunit beta	Copb1	5.23	3.55	1.61	1.09	0.68
Q99KQ4	Nicotinamide phosphoribosyltransferase	Nampt	7.07	4.82	70.69	48.15	0.68
Q8BP47	Asparagine--tRNA ligase, cytoplasmic	Nars1	9.80	6.69	2.06	1.41	0.68
Q9D0I9	Arginine--tRNA ligase, cytoplasmic	Rars1	8.41	5.75	1.75	1.20	0.68
Q56A10	Zinc finger protein 608	Znf608	7.96	5.66	7.08	5.03	0.71
Q0VGY8	Protein TANC1	Tanc1	5.18	3.78	51.76	37.82	0.73
Q8CEC0	Nuclear pore complex protein Nup88	Nup88	7.12	5.24	3.35	2.46	0.74
P47962	Large ribosomal subunit protein uL18	Rpl5	9.51	7.01	1.40	1.03	0.74
P97789	5'-3' exoribonuclease 1	Xrn1	14.19	10.47	141.91	104.71	0.74
Q9JKY0	CCR4-NOT transcription complex subunit 9	Cnot9	9.46	7.01	94.61	70.13	0.74
P17426	AP-2 complex subunit alpha-1	Ap2a1	10.80	8.05	2.51	1.87	0.75

Table S3. Panther analysis of subsets of enriched and depleted proteins in epoxomicin-treated cells.

GO ID	GO level	GO label	Fold enrichment	Raw pValue	FDR	Mapped genes
**The subset of proteins enriched in the epoxomicin-treated cells**						
**GO – Biological process**						
0006936	0	muscle contraction	58,77807	1,76E-05	0,038686	Myh1, Myh8, Myh4
0003012	1	muscle system process	57,64773	1,86E-05	0,020514	Myh1, Myh8, Myh4
**GO – Biological process**						
0000146	0	microfilament motor activity	81,01843	6,61E-06	0,003941	Myh1, Myh8, Myh4
0140657	1	ATP-dependent activity	11,48537	0,00036	0,035794	Myh1, Psmc2, Myh8, Myh4
0003774	1	cytoskeletal motor activity	31,55455	0,000113	0,016902	Myh1, Myh8, Myh4
0051015	0	actin filament binding	24,98068	1,78E-05	0,005319	Myh1, Cfl1, Myh8, Myh4
0003779	1	actin binding	18,33445	5,98E-05	0,011883	Myh1, Cfl1, Myh8, Myh4
0044877	1	protein-containing complex binding	9,217964	0,000166	0,019831	Myh1, Nipbl, Cfl1, Myh8, Myh4
**GO – Cellular component**						
0016459	0	myosin complex	107,0601	2,8E-06	0,00138	Myh1, Myh8, Myh4
0015629	1	actin cytoskeleton	18,33445	5,98E-05	0,014715	Myh1, Cfl1, Myh8, Myh4
0043232	3	intracellular non-membrane-bounded organelle	5,12096	8,24E-05	0,010134	Myh1, Mov10, Larp1, Sin3a, Fbf1, Cfl1, Myh8, Myh4
0043228	4	non-membrane-bounded organelle	5,12096	8,24E-05	0,008107	Myh1, Mov10, Larp1, Sin3a, Fbf1, Cfl1, Myh8, Myh4
0099080	0	supramolecular complex	11,43281	6,04E-05	0,00991	Myh1, Mov10, Larp1, Myh8, Myh4
0003779	1	actin binding	18,33445	5,98E-05	0,011883	Myh1, Cfl1, Myh8, Myh4
0044877	1	protein-containing complex binding	9,217964	0,000166	0,019831	Myh1, Nipbl, Cfl1, Myh8, Myh4
**The subset of proteins depleted in the epoxomicin-treated cells**						
**GO – Biological process**						
0016192	0	vesicle-mediated transport	6,354284	1,04E-05	0,022987	Ap3b1, Ap2a1, Ap1b1, Copb1, Vps35, Eps15, Dab2, Sec31a, Dnm1
**GO – Cellular component**						
0030120	0	vesicle coat	30,99107	0,000124	0,020335	Ap2a1, Copb1, Sec31a
0030117	1	membrane coat	33,40881	6,21E-06	0,003054	Ap2a1, Copb1, Eps15, Sec31a
0005622	3	Intracellular anatomical structure	1,630206	0,000284	0,027973	Rpl5, Cct6a, Nono, Asns, Vps35, Cep170, Gli1, Farsa, Dab2, Rpl6, Macf1, Ncoa3, Copb1, Smarcd2, Numa1, Dvl2, Ecpas, Cand1, Ptbp1, Nars1, Tacc2, Xpo7, Uba52, Cnot9, Nup88, Eps15, Sec31a, Dnm1, Dnm1l, Eif4a3, Ap2a1, Eif4a2, Kmt2c, Lrpprc, Znf608
0048475	2	coated membrane	33,40881	6,21E-06	0,001527	Ap2a1, Copb1, Eps15, Sec31a
0030662	1	coated vesicle membrane	26,76502	0,000193	0,023686	Ap2a1, Copb1, Sec31a

Table 3. Panther analysis of proteins from Table S1 and Table S2 listing Gene Ontology (GO) clusters for Biological process, Molecular function, and Cellular component categories. The GO level column represents the parental relationship between GO numbers.
